# Thyroid function during COVID-19 and post-COVID complications in adults: a systematic review

**DOI:** 10.3389/fendo.2024.1477389

**Published:** 2025-02-04

**Authors:** Anisha Panesar, Palma Gharanei, Natasha Khovanova, Lawrence Young, Dimitris Grammatopoulos

**Affiliations:** ^1^ Warwick Medical School, University of Warwick, Coventry, United Kingdom; ^2^ School of Engineering, University of Warwick, Coventry, United Kingdom; ^3^ Institute of Precision Diagnostics and Translational Medicine, University Hospitals Coventry and Warwickshire National Health Service (NHS) Trust, Coventry, United Kingdom

**Keywords:** cytokines, thyroid, COVID-19, long COVID, inflammation

## Abstract

The coronavirus disease 2019 (COVID-19) pandemic, caused by the severe acute respiratory syndrome coronavirus 2 (SARS-CoV-2) virus, has presented multifaceted health challenges. COVID-19 primarily targets the respiratory system but also affects multiple organ systems, including the endocrine system. Emerging evidence suggests interactions between thyroid function, the acute phase of COVID-19, and the prolonged symptoms known as post-COVID sequalae or long COVID. Several studies have reported that COVID-19 can induce thyroid dysfunction, leading to conditions such as thyroiditis and alterations in thyroid hormone levels. The mechanisms through which SARS-CoV-2 affects the thyroid include direct viral infection of thyroid cells, leading to viral thyroiditis, which causes inflammation and transient or sustained thyroid dysfunction, as well as an excessive systemic immune response (cytokine storm). This is associated with elevated levels of cytokines, such as IL-6, that disrupt thyroid function and lead to nonthyroidal illness syndrome (NTIS). Medications administered during the acute illness phase, such as corticosteroids and antiviral drugs, can also impact thyroid hormone actions. The involvement of the thyroid gland in long COVID, or postacute sequelae of SARS-CoV-2 infection, is an area not well defined, with potential implications for understanding and managing this condition. Persistent low-grade inflammation affecting thyroid function over time can lead to ongoing thyroiditis or exacerbate pre-existing thyroid conditions. Viral infections, including SARS-CoV-2, can trigger or worsen autoimmune thyroid diseases, such as Hashimoto’s thyroiditis and Graves’ disease. Long COVID may disrupt the hypothalamic–pituitary–adrenal (HPA) axis, which can, in turn, affect the hypothalamic-pituitary-thyroid (HPT) axis, leading to abnormal thyroid function. This review was designed to systematically capture recent literature on COVID-19-related thyroid dysfunction in the adult population, the prognostic consequences of thyroid dysfunction during COVID-19, and the effects of thyroid dysfunction on patients with long COVID. A comprehensive search of PubMed and EMBASE databases was conducted. The systematic review was performed based on the Preferred Reporting Items for Systematic Reviews and Meta-Analyses (PRISMA) statement. Study quality was assessed using the Critical Appraisal Skills Programme (CASP). A total of 53 studies met the inclusion criteria. The review summarises recent findings and provides an update of the current understanding of thyroid dysfunction in COVID-19-related spectrum of disorders, underscoring the complex nature of SARS-CoV-2 infection and its far-reaching impacts on human health.

## Introduction

Since the first case of the coronavirus disease 2019 (COVID-19), an extensive volume of literature has been published on its acute phase, caused by severe acute respiratory syndrome coronavirus 2 (SARS-CoV-2), as well as its chronic counterpart, long COVID or postacute COVID syndrome. Although COVID-19 is predominately known for affecting the respiratory system, it also presents nonpulmonary symptoms, such as cardiac abnormalities, liver disease, and endocrine dysfunction (1). Among the endocrine systems affected during COVID-19 infection, thyroid dysfunction is frequently recognised, resulting from both direct and indirect mechanisms that can disrupt thyroid homeostasis.

Thyroid function is tightly controlled by the hypothalamic–pituitary–thyroid axis (HPT), which regulates the secretion of thyrotropin-releasing hormone (TRH) and thyroid-stimulating hormone (TSH). These hormones induce the synthesis of thyroid hormones (namely 3,3′,5,5′-tetraiodo-l-thyroxine (T4) and 3,5,3′-triiodo-l-thyroinine (T3) triiodothyronine), which are then released into circulation. A series of negative feedback loops tightly regulate HPT axis activity (2). Moreover, the peripheral availability of T3 is controlled by deiodination of T4 and its conversion into T3 in the liver, kidneys, and muscles by deiodinase (DIO)1 and DIO2 enzymes, whereas DIO3 deiodinates T4 to produce reverse T3 (2).

Immune system regulation by thyroid hormones is well recognised, as they have been shown to influence leucocyte proliferation and migration, antibody production, and the release of cytokines, which can trigger immune responses against microbial or sterile insults. Effects in the opposite direction have also been described where the activation of inflammatory pathways and infection can affect the HPT axis and the downstream activity of thyroid hormones. This can occur indirectly through the action of cytokines such as interleukin (IL)-1, IL-6, and tumour necrosis factor (TNF)-α, which act on the hypothalamus, dampening TSH activity and resulting in decreased production of T3 and T4 (2). Additionally, indirect insults are also thought to play a role in autoimmune thyroid disease, as SARS-CoV-2 hyperactivates the immune response, leading to a consequent increase in IL-6 and T-cell helper (Th) lymphocytes, such as interferon (IFN)-γ-secreting Th1 cells and IL-17-secreting Th17 cells. These Th cells are produced in peripheral lymphocytes, and increased levels of these Th cells and their associated cytokines have been reported in cases of autoimmune thyroid disease, making it plausible that they could be involved the development of autoimmune disease through apoptotic pathways in thyroid follicular cells, leading to thyroid cell destruction (3).

**Figure 1:**
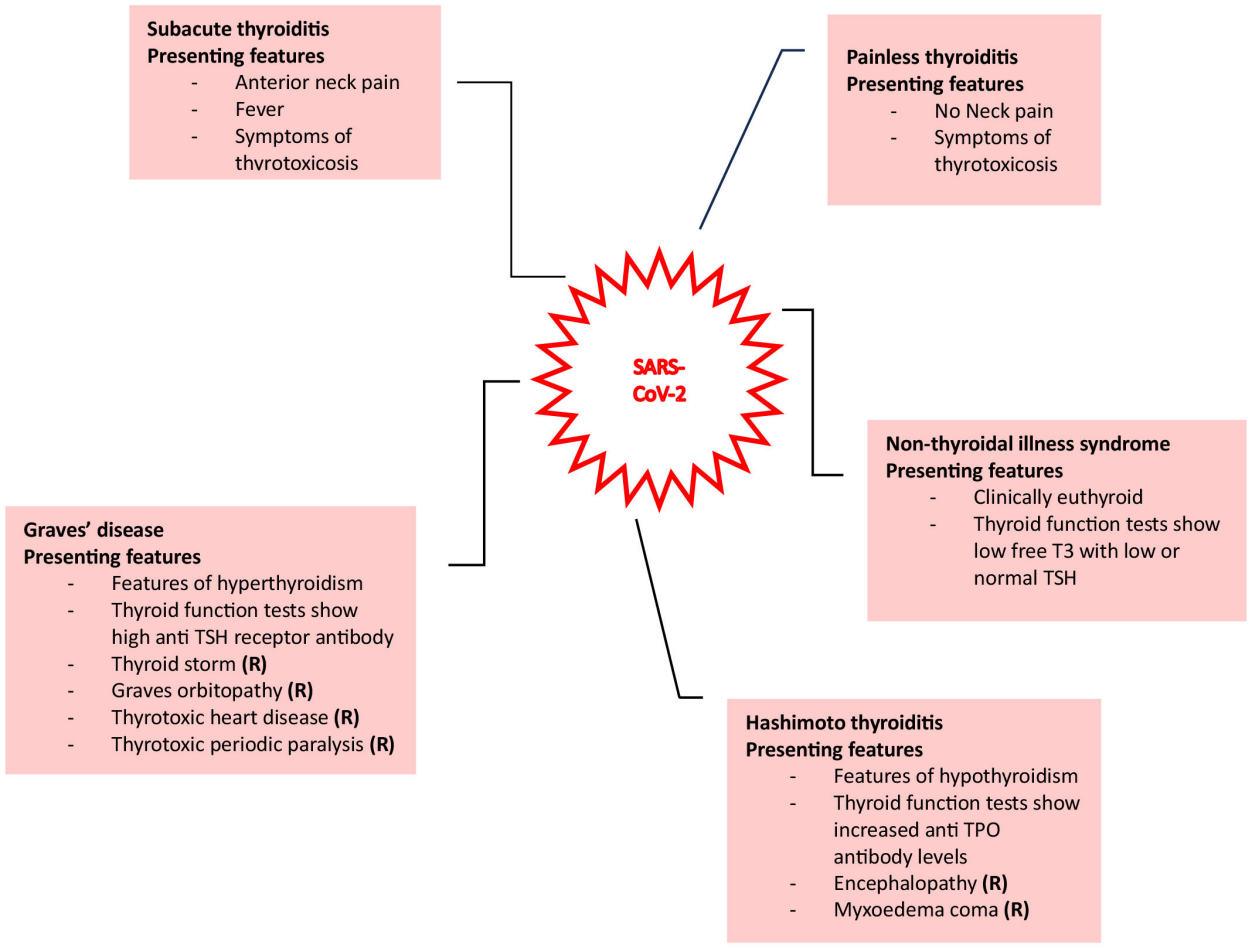
Various presentations of thyroid injury due to SARS-CoV-2 are shown here (1).

Alternatively, direct viral injury can occur, whereby infections such as SARS-CoV-2 and disorders like acute respiratory distress syndrome can cause destruction of thyrocytes. The manifestations of injury specifically linked to SARS-CoV-2 are displayed in **Figure 1**. The ability of the virus to exert these multisystem effects is thought to be due to the widespread expression of receptors for SARS-CoV-2, namely angiotensin-converting enzyme 2 (ACE2) and its coreceptor transmembrane protease serine 2 (TMPRSS2) (1). This has been shown in deceased patients due to COVID-19, where viral mRNA was detected in the blood, urine, and stool of these patients. Furthermore, immunohistochemical detection of SARS-CoV-2 viral proteins within thyroid tissue provides further evidence for the interaction of SARS-CoV-2 with ACE2 and TMPRSS2 (4–6). Additionally, multiple studies in patients with COVID-19 employing ultrasound identified markedly hypoechoic focal areas in thyroid tissue consistent with inflammation. These patients were later diagnosed with subacute or atypical thyroiditis. Piecing this evidence together, direct damage to the thyroid gland by SARS-CoV-2 is a plausible explanation for thyroid involvement during COVID-19.

Following the initial wave of disease in early 2020, chronic symptoms have been reported and investigated in survivors. In the disease setting now identified as long COVID, these symptoms are often serious and are thought to affect at least 10% of COVID-19 survivors. Fatigue, joint pain, “brain fog”, chest pain, low mood, cough, shortness of breath, headaches, and muscle pain are some of the symptoms that have been reported to be associated with long COVID (3, 7). The underlying pathogenesis of this syndrome is not completely understood; however, one hypothesis suggests that thyroid involvement, especially the transient phase of thyroid dysfunction in the convalescent period, remains an ongoing issue in some COVID-19 patients. Conversely, it is also thought that the immunological dysfunction from the virus can lead to increased antithyroid antibodies, resulting in autoimmune thyroid disease (8). Such pathological processes involve either autoantibodies to thyroid peroxidase (TPO-Ab) and thyroglobulin (Tg-Ab), leading to hypothyroidism, or TSH receptor autoantibodies (TSH-R Ab) causing hyperthyroidism (9).

A wide range of thyroid dysfunctions have been reported, with their symptoms described in **Table 1**. Concerns have been raised that thyroid abnormalities might be masked or remain unnoticed, as many symptoms overlap with the symptoms of COVID-19, such as intense fatigue and fever. Furthermore, the complications related to thyroid function can vary and, in extreme cases, can be life-threatening, such as subacute thyroiditis.

**Table 1:** Types of thyroid dysfunction identified in COVID-1 patients.

Thyroid Condition	Common Symptoms	Investigations	Treatment
**Subacute thyroiditis**	Characteristically, it has three phases: painful swelling of the thyroid gland, hypothyroidism, and euthyroidism. Typical symptoms, such as neck pain and persistent tachycardia, are key indicators (10).	Thyroid dysfunction present as thyrotoxicosis; most cases are antithyroid antibody negative. Ultrasound of the thyroid gland will show focal hypoechoic areas. Typically, thyroid scintigraphy shows less than 2% uptake of radioactive iodine within 24 h (3).	Self-limiting; however, beta-blockers are used for symptom relief. Nonsteroidal anti-inflammatory drugs are given in mild cases to reduce inflammation and pain. Prednisolone is administered to patients with severe pain (3).
**Painless thyroiditis**	A variation of thyroiditis with the absence of neck pain. Other symptoms are similar to those of subacute thyroiditis (1).	Thyroid dysfunction presents as thyrotoxicosis; most cases are antithyroid antibody negative. Ultrasound of the thyroid gland shows focal hypoechoic areas. Typically, thyroid scintigraphy shows low uptake of radioactive iodine in 24 h (1).	Beta blockers are used for symptom control. Glucocorticoids are administered in in severe cases (1).
**Graves’ disease**	An autoimmune condition causes immune cells to attack the thyroid, resulting in hyperthyroidism. It can also lead to thyroid eye disease and pretibial myxoedema. Graves’ disease involves increased thyroid hormone synthesis, release, and growth due to thyroid-stimulating hormone receptor antibodies (1).	Thyroid function will show thyrotoxicosis, and high levels of antithyroid-stimulating hormone receptor (TSH-R) antibodies will help confirm the diagnosis. A thyroid ultrasound will show diffuse enlargement of the thyroid gland, as well as increased vascularity. Thyroid scintigraphy will show a diffuse increase in the uptake of radioactive iodine (1).	Thionamides and beta blockers are administered (1).
**Hashimoto’s thyroiditis**	A common autoimmune condition caused by the destruction of the thyroid gland and the presence of either antithyroid peroxidase antibodies and/or antithyroglobulin antibodies. In the short term, it can present with fatigue, weight gain, dry skin, and constipation. It typically presents with neuromuscular symptoms, with or without a goitre (11).	Thyroid function shows primary hypothyroidism with high levels of antithyroid peroxidase antibodies to help confirm the diagnosis (1).	Levothyroxine is given (1).
**Non-thyroidal illness syndrome**	This syndrome presents with alterations in thyroid hormones and is seen in up to 70% of hospitalised patients with critical illness, e.g., after major surgery, sepsis and SIRS, COVID-19, and other viral illnesses. Clinically, patients are euthyroid (1).	Thyroid profile exhibits low T3, high levels of reverse T3 (rT3), low or normal T4, and low or normal thyroid-stimulating hormone (TSH) (1).	Self-limiting, but close supervision is needed (1).

Thyroid condition is identified in bold.

The global response to the pandemic is shifting towards disease prevention, with the implementation of widespread vaccination programmes and management of the long-term consequences of the disease. Therefore, a summary of the latest findings regarding thyroid dysfunction in COVID-19 could provide a timely update on the current understanding (1). The objectives of this review were to systematically evaluate recent literature on COVID-19-related thyroid dysfunction, the prognostic consequences of thyroid dysfunction during COVID-19, and the effects of thyroid dysfunction on patients with long COVID.

## Methods

### Search strategies and study selection

To develop the search strategy and review question, a Population, Intervention, Comparison, and Outcome (PICO) format (**Table 2**) was used.

**Table 2:** PICO format.

**Population**	Patients over the age of 18 years with COVID-19 and no history of thyroid disease
**Intervention**	Awareness of thyroid abnormalities in COVID-19 patients
**Comparison**	Patients without COVID-19 and a history of thyroid disease
**Outcomes**	Whether the presence of thyroid abnormalities in COVID-19 patients affects their outcomes and recovery

Bold: components of the clinical question.

A search was then carried out systematically on Medline and EMBASE, and papers in the English language published between January 2020 and April 2024 were included to ensure the results were accurately interpreted. The search strategy, shown in **Table 3**, was based on the following keywords/phrases: (COVID-19 OR coronavirus disease OR long COVID OR SARS-CoV-2 OR severe acute respiratory syndrome coronavirus 2) AND (subacute thyroiditis OR painless thyroiditis OR subacute lymphocytic thyroiditis OR silent thyroiditis OR Graves’ disease OR Hashimoto’s thyroiditis OR Hashimoto’s disease OR nonthyroidal illness syndrome OR euthyroid sick syndrome). These terms were used in order to ensure a broad search that covered all relevant literature. On the other hand, terms such as “autoimmune thyroiditis” were excluded to avoid ambiguity, providing more specific results and capture the most relevant studies. Specific quality control papers were also included to ensure the search retrieved appropriate publications. Furthermore, relevant secondary sources and grey literature were searched, as well as reference lists, to identify additional pertinent papers. EndNote was utilised to import and manage abstracts and full texts. Once the searches were completed, duplicates were removed. The title and abstracts, followed by the full texts, were then reviewed independently by two reviewers at separate times.

**Table 3:** Search strategy used for the databases Embase and Medline.

Database	Search strategy
**Embase**	1. COVID 19.mp. or exp coronavirus disease 2019/ 2. limit 1 to (English language and yr='2020 - Current*) 3. exp long COVID/or long covid.mp. 4. limit 3 to (English language and yr='2020 - Current*) 5. SARS-CoV-2.mp. or exp Severe acute respiratory syndrome coronavirus 2/ 6. limit 5 to (English language and yr="2020 -Current*) 7. 2 or 4 or 6 8. subacute thyroiditis.mp. or exp subacute thyroiditis/ 9. limit 8 to (English language and yr=*2020 - Current* 10. 7 and 9 11. painless thyroiditis.mp. 12. limit 11 to (English language and yr="2020 - Current*) 13. subacute lymphocytic thyroiditis.mp. 14. limit 13 to (English language and yr="2020 - Current') 15. silent thyroiditis.mp. 16. limit 15 to (English language and yr=*2020 - Current") 17. 12 or 14 or 16 18. 7 and 17 19. Graves disease.mp. or exp Graves disease/ 20. limit 19 to (English language and yr="2020 - Current') 21. 7 and 20 22. Hashimoto thyroiditis.mp. or exp Hashimoto disease/ 23. limit 22 to (English language and yr="2020 - Current*) 24. 7 and 23 25. non-thyroidal illness syndrome.mp. or exp euthyroid sick syndrome/ 26. limit 25 to (English language and yr=*2020 - Current') 27. 7 and 26
**Medline**	1. COVID 19.mp. or exp COVID-19/ 2. limit 1 to (English language and yra"2020 - Current*) 3. long covid.mp. or exp Post-Acute COVID-19 Syndrome/ 4. limit 3 to (English language and yr="2020 -Current") 5. long covid.mp. or exp Post-Acute COVID-19 Syndrome/ 6. SARS-CoV-2.mp. or exp SARS-CoV-2/ 7. limit 6 to (English language and yr="2020 - Current") 8. 2 or 4 or 7 9. subacute thyroiditis.mp. 10. limit 9 to (English language and yr="2020 - Current") 11. 8 and 10 12. painless thyroiditis.mp. 13. limit 12 to (English language and yr="2020 - Current*) 14. subacute lymphocytic thyroiditis.mp. 15. limit 14 to (English language and yra"2020 - Current*) 16. silent thyroiditis.mp. 17. limit 16 to (English language and yr="2020 - Current*) 18. 13 or 15 or 17 19. 8 and 18 20. Graves disease.mp. or exp Graves Disease/ 21. limit 20 to (English language and yr=*2020 - Current*) 22. 8 and 21 23. Hashimoto thyroiditis.mp. or exp Hashimoto Disease/ 24. limit 23 to (English language and yre"2020 - Current*) 25. 8 and 24 26. non-thyroidal illness syndrome.mp. or exp Euthyroid Sick Syndromes/ 27. limit 26 to (English language and yr«'2020 - Current') 28. 8 and 272

Database searched is identified in bold.

The full search strategy is shown in **Table 3**.

### Inclusion/Exclusion criteria

Case studies and reports, observational studies, retrospective studies, prospective studies describing the clinical features and outcomes of thyroid disease, including subacute thyroiditis, painless thyroiditis, silent thyroiditis, Graves’ disease, Hashimoto’s thyroiditis, or nonthyroidal illness syndrome, were included. Papers describing patients who were pregnant, those in the paediatric population (specifically under 18 years of age), or those with a previous history of thyroid disease, as well as letters/comments to the editor, were excluded from this review.

### Case definition and diagnostic investigations

Only papers with positive COVID-19 PCR tests, confirming COVID-19 infection, were included in this review; in contrast, cases speculated to be COVID-19 were not considered further.

In terms of thyroid dysfunction investigations, in the studies included in the review, the diagnosis of subacute thyroiditis was made on thyroid function tests suggestive of thyrotoxicosis, thyroid ultrasonography showing focal hypoechoic areas, and thyroid scintigraphy showing low or absent radiotracer uptake. Painless thyroiditis would show the same investigation results as subacute thyroiditis, but in the absence of pain.

The diagnosis of Graves’ disease was based on the presence of TSH-R Ab or thyroid-stimulating immunoglobulins (12), as well as the combination of supressed TSH levels and increased free T4 levels. However, in some cases, the diagnosis of thyrotoxicosis was based on raised levels of T3, but not T4, and undetectable TSH (T3-induced thyrotoxicosis).

Diagnosis of Hashimoto’s thyroiditis causing hypothyroidism was based on low or normal serum free T4 and increased serum TSH levels, along with the presence of high levels of thyroid peroxidase (TPO) autoantibodies. Additionally, thyroid ultrasonography would show an enlarged thyroid gland with a hypoechoic, diffusely heterogeneous echotexture which hypoechoic micronodules.

Finally, in the studies mentioned, the diagnosis of nonthyroidal illness syndrome was based on normal serum levels of TSH but reduced serum levels of free triiodothyronine (fT3).

### Data extraction

Data extraction from the included studies was organised by author, publication year, title, country, study design, number of patients included, patient data (including the age, sex, and severity of COVID-19), and thyroid characteristics. This was analysed independently by two authors and is displayed in **Supplementary Table 1**.

### Study quality assessment

A critical appraisal was completed using the Critical Appraisal Skills Programme (CASP), (https://casp-uk.net/checklists/casp-systematic-review-checklist-fillable.pdf). This was used to assess the quality of the studies included, as shown in **Tables 4**–**6**.

**Table 4:** Risk of bias assessment.

Studies	Publication bias	Generalisability bias	Self-selectionbias	Attrition bias	Reporting bias	Confirmation bias	Confounding bias	Other sources of bias
Baldelli et al. (13)	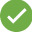	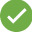	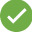	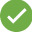	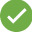	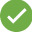	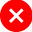	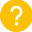
Álvarez Martín et al. (14)	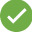	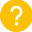	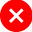	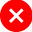	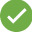	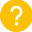	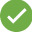	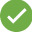
Mehmood et al. (15)	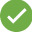	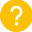	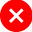	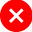	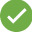	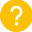	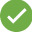	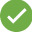
Brancatella et al. (16)	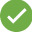	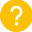	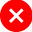	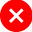	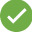	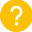	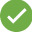	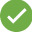
Asfuroglu Kalkan and Ates (17)	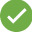	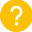	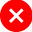	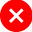	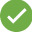	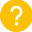	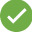	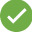
Batman et al. (18)	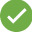	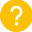	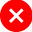	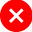	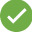	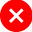	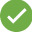	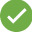
Arora et al. (19)	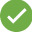	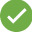	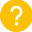	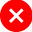	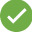	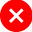	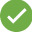	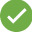
Shermetaro and Bushman (20)	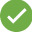	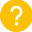	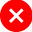	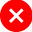	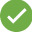	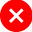	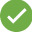	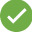
Henke et al. (21)	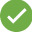	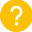	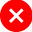	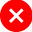	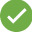	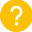	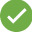	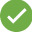
Lee et al. (11)	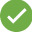	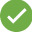	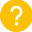	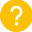	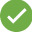	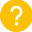	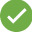	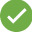
Muller et al. (22)	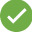	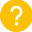	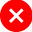	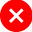	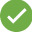	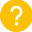	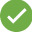	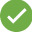
Franca et al. (23)	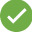	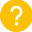	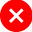	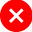	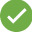	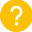	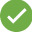	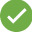
Nham et al. (24)	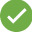	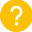	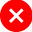	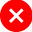	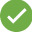	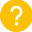	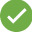	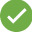
Peng et al. (25)	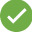	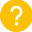	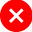	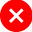	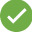	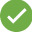	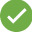	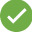
Boyle and Mullally (26)	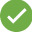	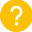	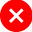	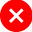	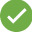	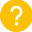	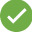	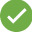
Lee et al. (11)	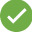	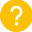	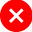	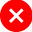	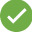	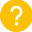	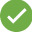	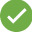
Rossini et al. (27)	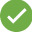	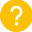	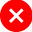	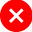	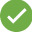	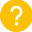	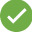	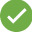
Feghali et al. (28)	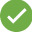	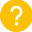	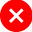	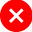	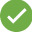	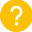	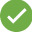	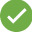
Knack et al. (29)	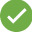	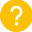	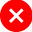	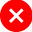	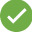	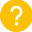	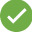	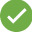
Lui et al. (1)	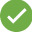	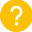	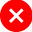	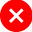	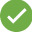	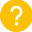	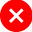	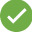
Millan et al. (30)	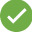	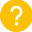	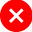	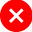	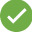	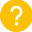	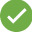	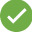
Dolkar et al. (31)	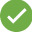	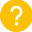	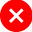	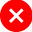	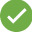	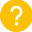	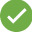	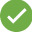
Sousa et al. (32)	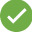	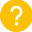	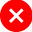	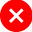	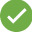	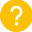	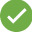	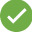
A.M Urbanovych 2021	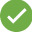	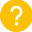	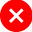	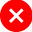	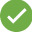	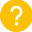	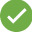	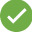
Asaf Harris, MD2020	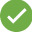	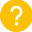	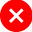	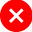	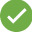	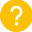	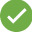	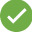
Vassiliadi et al. (12)	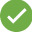	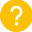	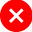	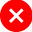	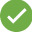	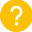	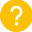	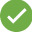
Swistek et al. (33)	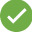	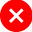	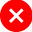	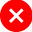	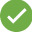	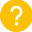	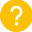	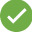
Sparano et al. (34)	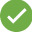	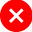	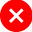	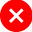	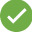	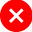	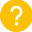	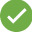
Okwor et al. (35)	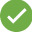	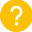	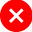	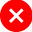	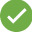	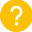	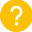	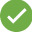
Gong et al. (36)	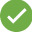	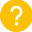	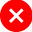	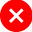	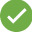	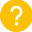	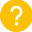	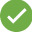
Schwarz et al. (37)	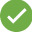	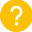	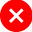	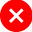	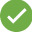	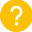	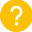	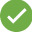
Campos-Barrera et al. (38)	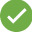	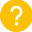	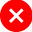	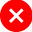	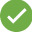	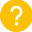	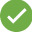	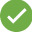
Sohrabpour et al. (39)	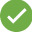	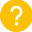	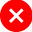	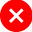	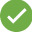	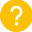	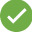	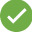
Akshay Khatri 2021	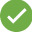	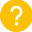	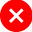	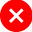	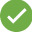	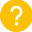	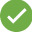	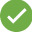
Whiting et al. (40)	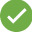	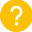	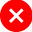	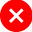	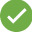	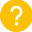	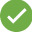	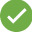
Davoodi et al. (41)	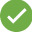	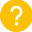	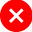	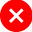	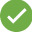	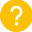	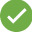	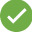
Ruggeri et al. (42)	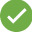	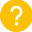	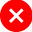	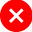	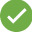	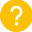	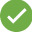	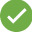
Brancatella et al. (16)	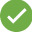	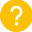	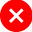	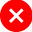	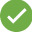	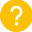	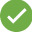	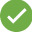
Hajosi-Kalcakosz et al. (43)	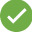	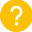	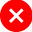	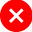	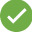	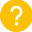	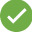	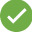
Elawady et al. (44)	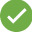	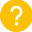	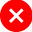	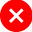	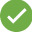	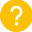	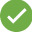	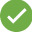
Tjonnfjord et al. (45)	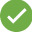	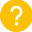	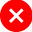	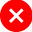	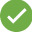	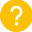	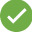	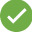
Chong et al. (46)	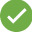	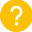	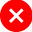	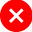	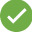	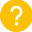	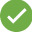	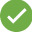
Sato et al. (47)	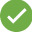	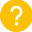	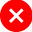	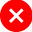	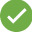	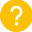	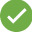	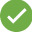
Al-Shammaa and Abdlkadir (48)	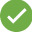	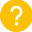	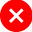	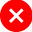	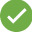	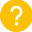	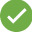	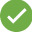
Gezer and Ecin (49)	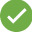	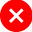	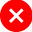	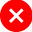	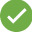	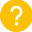	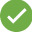	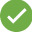
Ahn et al. (50)	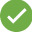	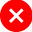	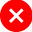	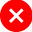	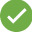	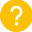	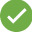	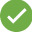
Edwards and Hussain (51)	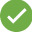	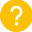	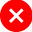	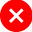	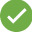	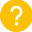	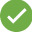	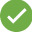
Mondal et al. (52)	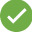	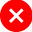	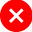	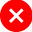	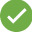	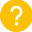	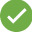	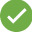
De Souza et al. (53)	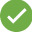	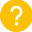	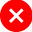	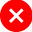	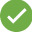	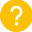	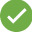	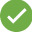
Lui et al. (1)	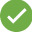	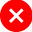	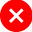	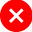	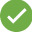	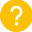	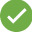	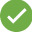
Zou et al. (54)	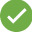	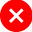	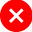	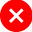	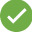	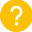	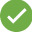	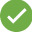
Wang et al. (55)	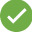	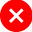	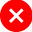	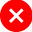	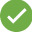	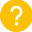	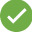	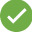
Georgios Tsivgoulis et al. (56)	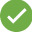	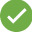	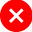	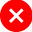	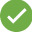	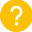	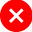	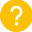
Vesselina Yanachkova and Radiana (8)	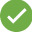	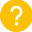	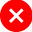	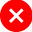	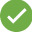	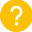	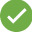	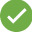

Key: 
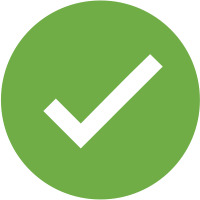
 Low risk; 
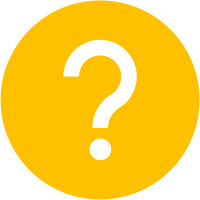
 Not Clear; 
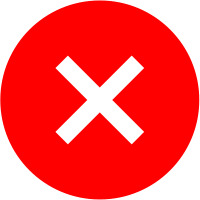
 High risk.

**Table 5:** Summary of JBL checklist for case reports.

Author and publication year	Q1	Q2	Q3	Q4	Q5	Q6	Q7	Q8	Critical appraisal score
Álvarez Martín et al. (2021)(14)	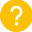	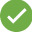	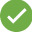	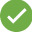	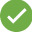	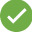	N/A	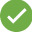	6/7
Mehmood et al. (2020)(15)	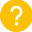	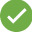	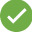	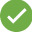	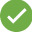	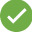	N/A	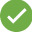	6/7
Brancatella et al. (2020a)(16)	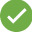	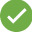	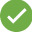	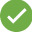	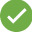	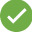	N/A	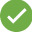	7/7
Asfuroglu Kalkan and Ates (2020)(17)	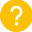	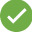	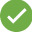	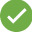	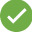	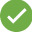	N/A	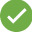	6/7
Henke et al. (2023)(21)	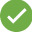	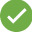	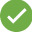	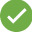	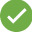	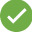	N/A	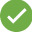	7/7
Franca et al. (2023)(23)	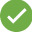	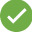	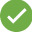	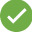	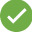	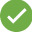	N/A	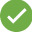	7/7
Nham et al. (2023)(24)	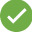	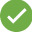	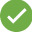	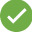	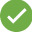	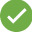	N/A	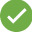	7/7
Shermetaro and Bushman (2023)(20)	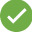	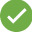	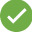	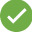	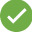	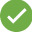	N/A	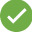	7/7
Boyle and Mullally (2023)(26)	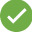	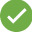	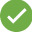	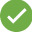	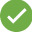	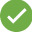	N/A	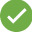	7/7
Lee et al. (2023)(11)	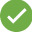	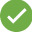	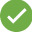	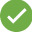	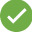	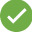	N/A	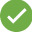	7/7
Feghali et al. (2021)(28)	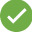	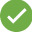	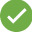	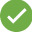	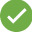	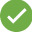	N/A	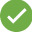	7/7
Knack et al. (2021)(29)	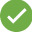	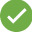	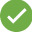	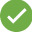	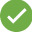	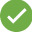	N/A	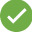	7/7
Millan et al. (2020)(30)	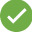	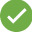	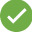	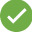	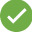	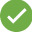	N/A	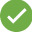	7/7
Dolkar et al. (2022)(31)	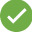	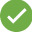	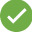	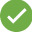	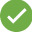	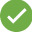	N/A	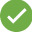	7/7
Sousa et al. (2022)(32)	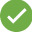	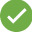	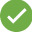	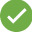	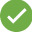	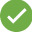	N/A	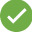	7/7
Urbanovych et al. (2021)(57)	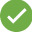	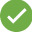	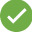	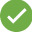	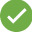	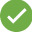	N/A	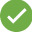	7/7
Harris and Al Mushref (2021)(58)	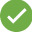	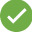	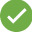	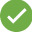	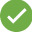	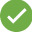	N/A	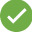	7/7
Campos-Barrera et al. (2020)(38)	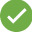	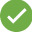	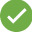	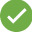	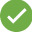	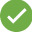	N/A	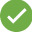	7/7
Sohrabpour et al. (2021)(39)	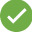	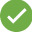	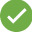	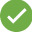	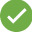	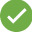	N/A	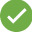	7/7
Khatri et al. (2021)(59)	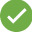	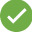	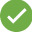	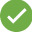	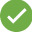	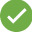	N/A	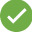	7/7
Whiting et al. (2021)(40)	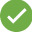	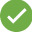	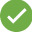	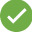	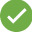	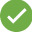	N/A	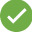	7/7
Davoodi et al. (2021)(41)	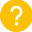	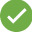	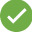	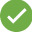	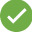	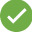	N/A	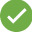	6/7
Ruggeri et al. (2021)(42)	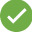	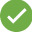	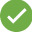	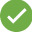	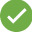	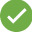	N/A	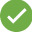	7/7
Brancatella et al. (2020a)(16)	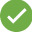	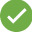	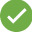	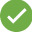	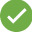	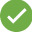	N/A	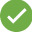	7/7
Hajosi-Kalcakosz et al. (2022)(43)	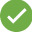	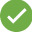	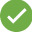	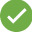	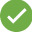	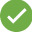	N/A	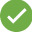	7/7
Elawady et al. (2022)(44)	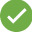	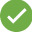	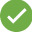	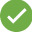	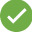	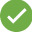	N/A	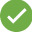	7/7
Tjonnfjord et al. (2021)(45)	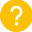	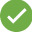	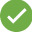	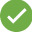	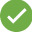	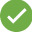	N/A	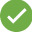	6/7
Chong et al. (2021)(46)	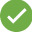	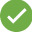	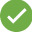	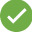	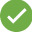	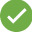	N/A	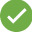	7/7
Sato et al. (2021)(47)	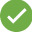	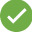	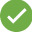	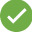	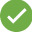	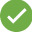	N/A	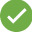	7/7
Al-Shammaa and Abdlkadir (2022)(48)	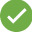	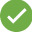	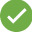	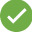	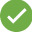	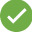	N/A	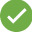	7/7
Edwards and Hussain (2021)(51)	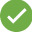	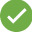	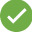	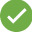	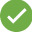	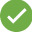	N/A	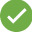	7/7
De Souza et al. (2022)(53)	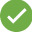	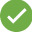	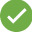	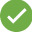	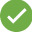	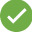	N/A	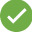	7/7

Key: 
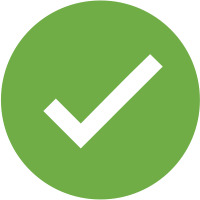
 Yes; 
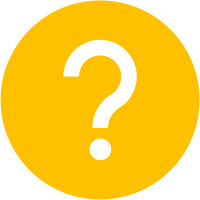
 Not clear; N/A, Not Applicable.

**Table 6:** Summary of the CASP checklist for quantitative studies; Q7 and 8 omitted, as the results of the studies are provided in **Supplementary Table 1**.

Author and publication year	Q1	Q2	Q3	Q4	Q5(A)	Q5(B)	Q6(A)	Q6(B)	Q9	Q10	Q11	Q12	Critical appraisal score
Baldelli et al. (2021)(13)	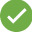	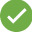	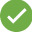	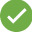	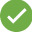	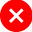	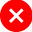	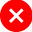	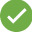	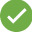	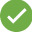	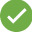	9/12
Batman et al. (2023)(18)	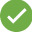	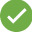	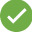	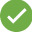	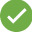	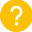	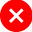	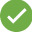	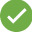	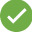	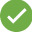	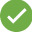	10/12
Arora et al. (2022)(19)	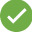	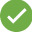	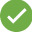	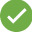	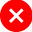	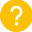	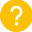	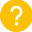	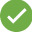	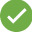	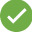	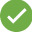	8/12
Lee et al. (2023)(11)	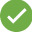	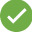	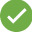	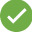	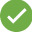	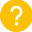	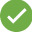	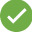	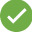	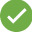	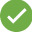	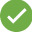	11/12
Muller et al. (2023)(22)	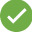	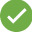	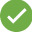	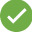	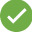	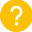	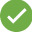	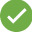	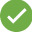	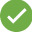	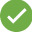	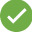	11/12
Peng et al. (2023)(25)	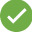	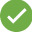	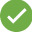	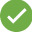	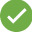	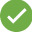	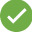	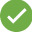	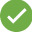	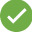	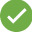	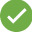	12/12
Rossini et al. (2023)(27)	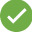	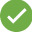	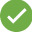	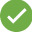	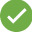	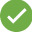	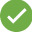	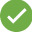	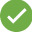	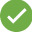	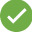	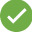	12/12
Lui et al. (1)	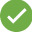	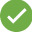	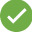	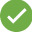	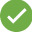	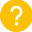	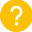	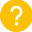	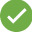	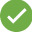	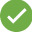	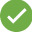	9/12
Vassiliadi et al. (2021)(12)	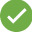	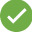	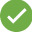	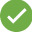	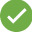	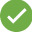	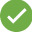	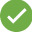	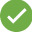	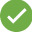	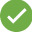	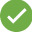	12/12
Swistek et al. (2022)(33)	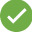	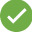	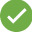	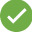	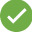	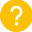	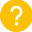	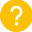	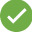	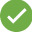	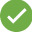	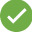	9/12
Sparano et al. (2022)(34)	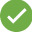	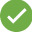	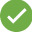	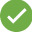	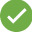	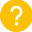	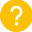	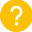	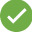	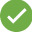	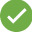	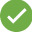	9/12
Okwor et al. (2021)(35)	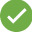	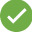	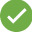	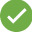	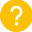	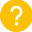	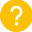	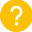	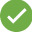	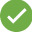	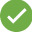	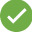	8/12
Gong et al. (2021)(36)	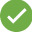	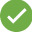	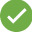	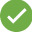	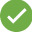	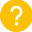	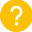	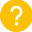	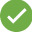	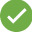	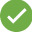	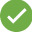	9/12
Schwarz et al. (2021)(37)	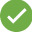	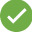	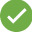	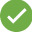	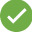	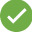	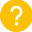	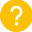	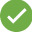	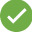	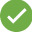	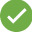	10/12
Gezer and Ecin (2022)(49)	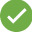	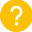	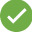	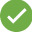	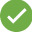	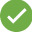	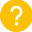	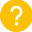	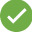	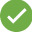	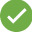	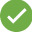	9/12
Ahn et al. (2021)(50)	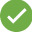	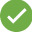	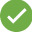	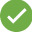	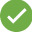	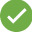	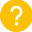	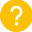	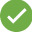	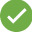	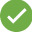	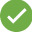	10/12
Mondal et al. (2023)(52)	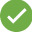	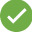	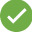	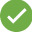	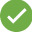	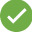	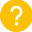	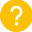	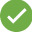	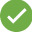	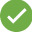	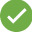	10/12
Lui et al. (1)	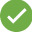	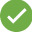	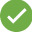	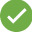	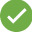	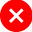	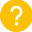	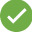	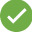	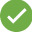	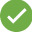	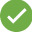	10/12
Zou et al. (2020)(54)	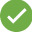	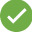	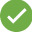	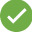	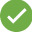	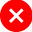	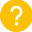	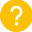	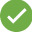	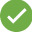	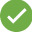	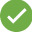	9/12
Wang et al. (2020)(55)	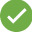	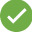	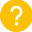	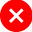	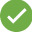	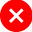	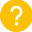	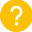	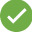	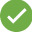	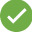	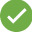	7/12
Vesselina Yanachkova and Radiana (2023)(8)	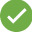	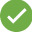	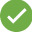	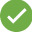	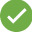	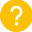	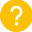	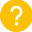	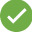	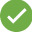	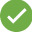	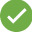	9/12

Key: 
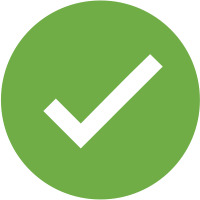
 Low risk; 
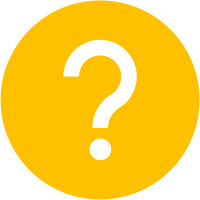
 Not Clear; 
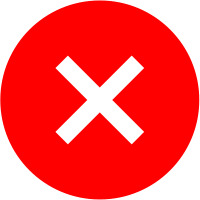
 High risk.

### Risk of bias assessment

A risk of bias assessment was completed using a customised risk of bias tool, due to the nature of each study, as shown in **Table 7**.

**Table 7:** Summary of the CASP checklist for case control studies; Q7 and Q8 omitted, as results of the studies are provided in **Supplementary Table 1**.

Author and publication year	Q1	Q2	Q3	Q4	Q5	Q6(a)	Q6(b)	Q9	Q10	Q11
Georgios Tsivgoulis et al. (2021)(56)	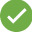	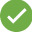	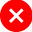	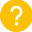	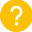	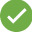	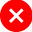	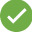	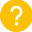	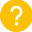

Key: 
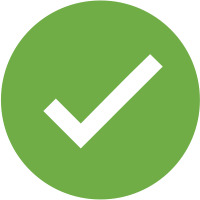
 Low risk; 
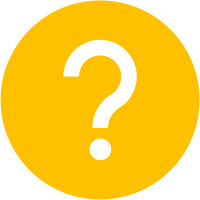
 Not Clear; 
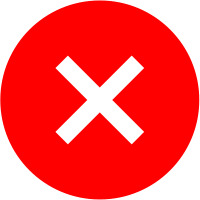
 High risk.

### Synthesis of extracted evidence

Narrative synthesis was used to critically review the latest advancements related to thyroid dysfunction and COVID-19.

## Results

### Search results and studies characteristics

**Figure 2:**
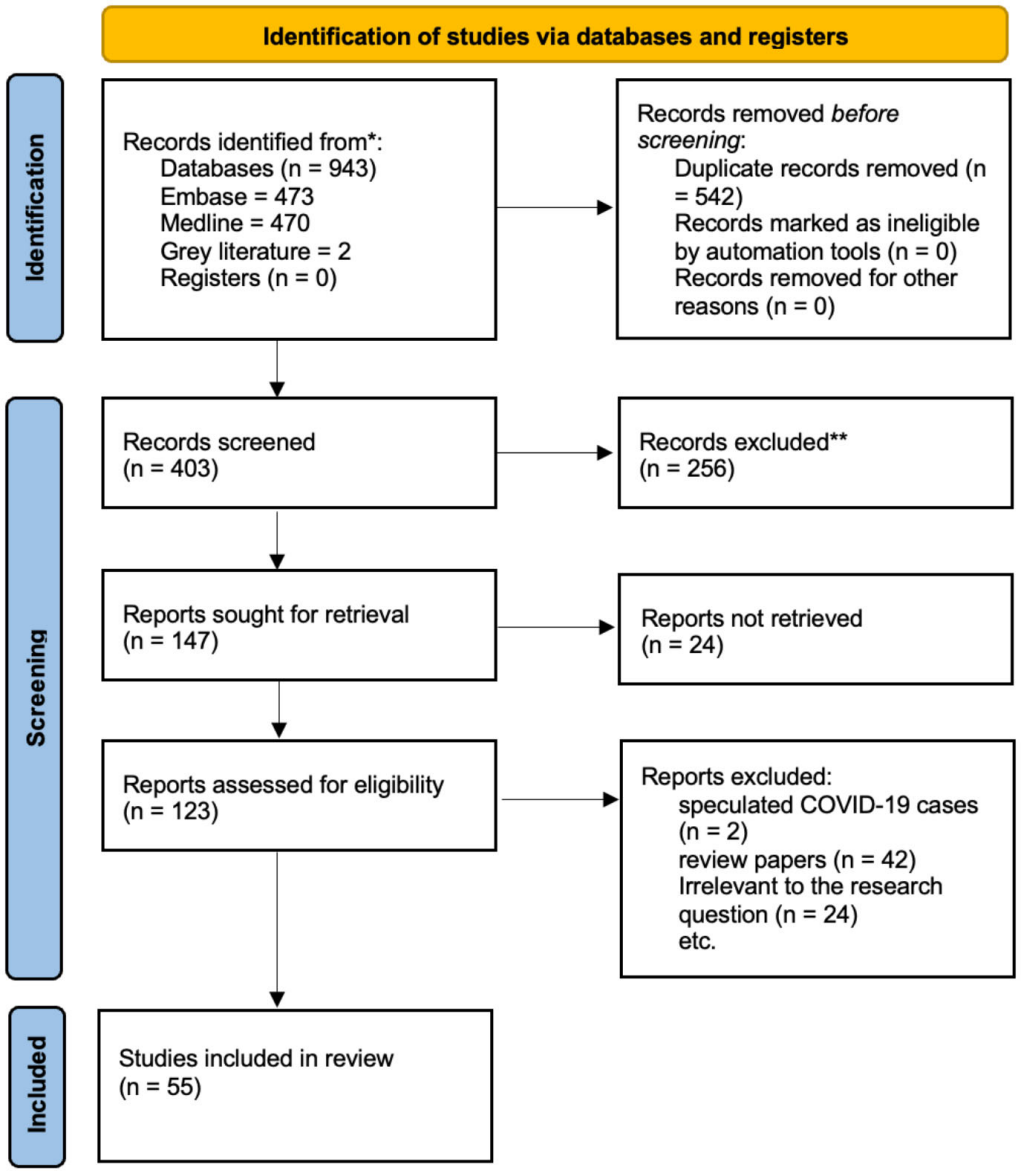
The PRISMA flowchart, depicting the searches of registers and databases, is shown here.

The total number of studies identified from the searches was 53 studies. A PRISMA flow diagram, displayed in **Figure 2**, shows the included and excluded articles, as well as the reasons for exclusion. The most common study design was case studies, and details of the findings of each study can be found in **Supplementary Table 1**.

### Case reports

The total number of patients reported with thyroid dysfunction from the case studies was 43, from 31 case studies. Subacute thyroiditis was the most commonly reported, with 64% (28 patients) of patients being diagnosed with the condition (9, 15, 17, 21, 24, 38, 39, 41–44, 59, No. 77; 28, 45–48, 53, 60. Graves’ disease was reported in 21% (nine patients) of patients (20, 23, 24, 26, 28, 32, 57, 58). Hashimoto’s thyroiditis was reported in 9% (four patients) of patients (11, 28, 29), and 5% (two patients) were diagnosed with painless thyroiditis (30, 31). These cases have been described from multiple countries, including Spain, the USA, Italy, Turkey, Switzerland, Korea, Singapore, and Brazil. From the case reports, most patients were women (66% of patients [29]), and the age range was between 18 and 81.

All patients suspected of having subacute thyroiditis presented with symptoms such as neck pain and tenderness and hypothyroidism-related fatigue. Typical symptoms, investigations, and management are described in **Table 1**. One patient experienced thyrotoxicosis and was later diagnosed with subacute thyroiditis after further investigation. However, 2 months later, a diagnosis of Graves’ disease was made due to fluctuating thyroid function tests (24). Two patients with painless thyroiditis exhibited the same pattern of investigations, showing signs of thyrotoxicosis, but without neck pain or tenderness (30, 31).

Among the patients who developed Graves’ disease, one also experienced eye irritation in addition to the typical symptoms outlined in **Table 1**. Patients with untreated Graves’ disease can deteriorate into life-threatening illness, as shown in two patients who experienced a thyroid storm, defined by Burch–Warsofsky point scale (BWPS) scores of 45 and 55, respectively. Two additional patients had BWPS scores of 35 and 40, indicating an impending thyroid storm. All the patients were treated with medication until they became euthyroid. Once they reached this point, they were continued on a maintenance dose (20, 26, 51).

Patients who experienced Hashimoto’s thyroiditis following COVID-19 all reported fatigue as one of their symptoms. Other characteristic symptoms, investigative approaches, and management are shown in **Table 1**.

### Retrospective studies

Altogether, the total number of retrospective studies included in this review was 12. Three focused on subacute thyroiditis post-COVID-19 (11, 18, 52), five focused on general thyroid dysfunction post COVID-19 (13, 19, 49, 50, 55), and four focused on nonthyroidal illness syndrome (NTIS) post-COVID-19 (33, 36, 37, 54).

One study focusing on subacute thyroiditis, which included a sample size of 98 patients with subacute thyroiditis after COVID-19 infection, found that the most common symptom was neck pain and tenderness, with the average time from diagnosis of subacute thyroiditis following COVID-19 being 21 days (18). Another study with a sample size of 160 found that the mean onset of subacute thyroiditis following COVID-19 was 23.8 days, whereas painless thyroiditis was reported more frequently soon after COVID-19 recovery, with a mean onset of around 10.6 days following COVID-19 infection (52). The study by Lee et al. (11), with a large sample size of 407,427, found a higher incidence rate of subacute thyroiditis (SAT) in COVID-19 patients compared to non-COVID-19 patients.

Among all five studies focusing on general thyroid dysfunction, all included patients who were diagnosed with or had clinical markers of NTIS (13, 19, 49, 50, 55). One study with a sample size of 119 identified NTIS in 18.5% of all patients, which was the most common abnormality found, followed by subclinical thyrotoxicosis, diagnosed in 14.3% of patients (50). Another study investigated 102 patients and reported similar findings, with NTIS diagnosed in 58.8% of patients (19). This was also demonstrated in the study by Baldelli et al. (13), where 28 out of 46 patients had low serum fT3 levels and were diagnosed with NTIS. The study by Gezer and Ecin (49) found that a low TSH level was significantly correlated with increased length of hospital stay and clinical severity, with 110 patients diagnosed with subclinical hypothyroidism. Furthermore, 116 patients from this study had low T3 levels, indicating that more than half of the sample size had clinical markers suggestive of NTIS. This was based on a sample size of 201.

The paper by Arora et al. (19) further demonstrated that low fT3 levels, which are common in NTIS, were shown to have a significant relationship with increased severity of COVID-19 and mortality. This is reiterated in all four studies focusing specifically on NTIS following COVID-19. For example, the paper by Swistek et al. (33) found that 28 out of 82 patients who developed NTIS died during hospitalisation, compared to 15 out of 133 patients without thyroid dysfunction during hospitalisation for COVID-19. In the paper by Gong et al. (36), the mortality rate was shown to be significantly higher in the low FT4 group of patients, and low TSH levels were shown to be independently related to 90-day mortality. Schwarz et al. (37) similarly found fT3 to be a significant independent predictor of mortality, which was echoed in the study by Zou et al. (54), which reported a significantly higher prevalence of severe events in patients diagnosed with euthyroid sick syndrome, also known as NTIS. Comparably, the study by Wang et al. (55) concluded that abnormal thyroid dysfunction was more common in severe cases of COVID-19 (47 patients out of a total of 52) than in mild or moderate cases of COVID-19 (16 patients out of a total of 52).

### Prospective studies

Four of the papers included in this review were prospective studies. One study, exploring the increased prevalence of autoimmune thyroid disease after COVID-19 in a patient cohort of 494, found that the prevalence doubled in COVID-19 survivors compared to controls (27). Another study, with a patient size of 506, focusing on NTIS after COVID-19 and its impact on patient outcomes, demonstrating a 3.5-fold increased risk of diminished survival in patients with lower fT3 levels (34). Lui et al. (1) studied 191 COVID-19 survivors to investigate thyroid disease, finding that 2% had new-onset abnormal thyroid function tests. Follow-up studies from the same group described the most common symptoms experienced by patients with thyroid dysfunction post-COVID-19.

## Discussion

### Thyroid involvement in COVID-19

The relationship between COVID-19 infection and its impact on thyroid gland function and activity is not yet fully understood. Emerging studies suggest both direct viral injury through pathophysiological mechanisms (3) and indirect systemic and generalised inflammatory responses (5) targeting the thyroid gland.

**Figure 3:**
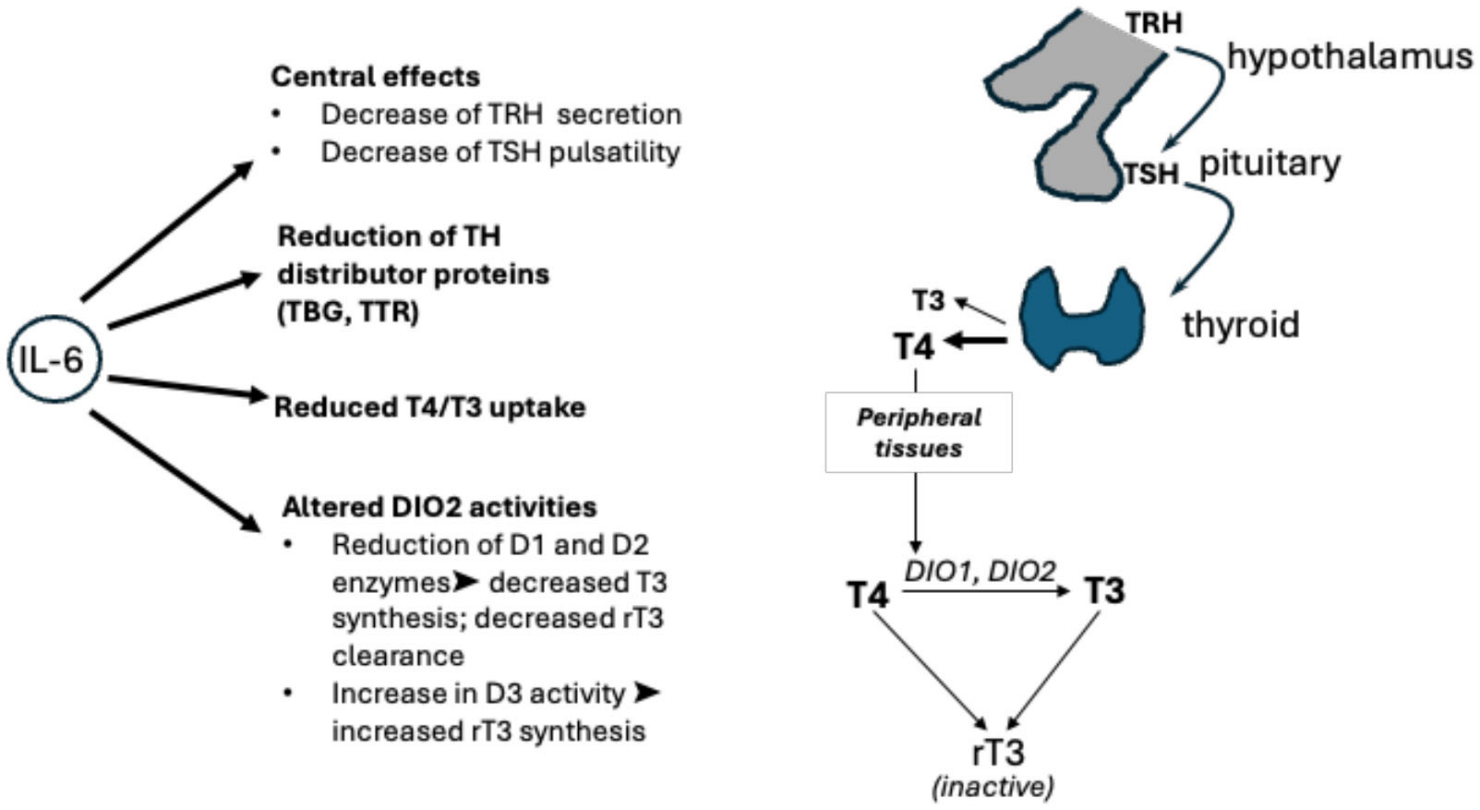
Hypothalamic–pituitary–thyroid axis: interactions between TRH, TSH, T3, and T4 along the hypothalamic–pituitary–thyroid axis are shown, alongside conversion by deiodinases 1, 2, and 3. The effects of interleukin (IL)-6 on thyroid hormone levels as shown on the left. The inflammatory cytokine acts on the hypothalamus and pituitary gland of the brain, on levels of free hormones, on transporters (TBG, TTR), and on cellular deiodinases. TBG, thyroxine binding globulin; TRH, thyrotropin releasing hormone; TSH, thyroid stimulating hormone; TTR, transthyretin.

A significant number of publications focus on the role of the cytokine storm, which causes indirect injury due to an uncontrolled systemic inflammatory response. In many patients, this is associated with NTIS (also known as euthyroid sick syndrome), which is commonly present in up to 70% of hospitalised patients that have critical illnesses, e.g., after major surgery, sepsis, SIRS, COVID-19, and other viral illnesses (61). The main cytokines involved in the COVID-19-driven inflammatory response have been identified as interleukin 6 and interleukin 1b (IL-1b) (62). These cytokines have also been found to act on the hypothalamic–pituitary–thyroid axis, altering TSH responsiveness to circulating low fT3 levels and resulting in inappropriately low/normal levels of serum TSH (**Figure 3**). Activation of cytokines involves a series of inflammatory signalling pathways, including nuclear factor kappa-light-chain enhancer of activated B cells (NFkB). During inflammation, NFkB has been demonstrated to upregulate deiodinase 2 (DIO2). In contrast DIO1 is downregulated with IL-1b stimulation. DIO1 has been closely linked to the pathogenesis of NTIS/ESS, as it has been shown to be greatly reduced during illness, resulting in low T3 levels. DIO1 and DIO2 mediate the peripheral conversion of circulating T4 into the biologically active hormone T3. Another deiodinase isoform, D3, is known as the key player in inactivating thyroid hormones, as it converts T4 and T3 into the biologically inactive rT3 (63).

The development of autoimmune thyroid diseases post-COVID-19 is not completely understood. A proposed hypothesis suggests that COVID-19 can trigger latent hyperactive reactions or instigate new-onset autoimmune disease. This reaction is thought to be linked to the “cytokine storm”, whereby the “viral infection-mediated hyperinflammatory condition” could activate the immune system, leading to autoimmune thyroid diseases such as Graves’ disease and Hashimoto’s thyroiditis. The “cytokine storm”, which leads to the overproduction of proinflammatory cytokines, is responsible for an overresponsive, uncontrolled reaction of the immune, coagulation, inflammatory, and complement systems, as well as multiorgan failure. This pathological setting can lead to death in severe cases, as the “cytokine storm” causes epithelial and endothelial cell injury, resulting in vascular leakage and increased permeability, ultimately leading to end-organ damage. In autoimmune thyroid disease, there have been reports of an increase in specific Th and cytokines. Imbalances in Th1 and Th2, along with increases in Th1 and Th17 in peripheral lymphocytes, as well as elevated levels of cytokines such as IL-17, IL-21, IL-23, IFN-γ, and TNF-α, have been observed in the serum of COVID-19 patients, further linking autoimmune disease to hyperstimulation of the immune system from COVID-19 (3).

Another potential explanation for the development of autoimmune thyroid disease post-COVID-19 is molecular mimicry. Research has identified several SARS-CoV-2 peptide sequences as homologous to human peptide sequences, resulting in cross-reactions between newly produced antibodies against the virus and the body’s self-antigens. Proteins identified in the SARS-CoV-2 proteome, such as the spike protein, membrane protein, and nucleoprotein, share similar peptide sequences with TPO, leading to this cross-reactions and, subsequently, thyroid autoimmunity. Transcriptional changes within immune genes of the thyroid gland have also been proposed as a plausible hypothesis for the pathogenesis of autoimmune thyroid disease in COVID-19. Studies have demonstrated a stronger innate immune response, which increases macrophage activity. This response could lead to inflammation and damage to thyroid tissue, resulting in symptoms frequently observed in SAT (7).

### Subacute thyroiditis post-COVID-19 illness

SAT, also known as de Quervain thyroiditis, is more commonly triggered by viral infections, specifically those of the upper respiratory tract, such as Coxsackie viruses and coronaviruses (1). Since the start of COVID-19 pandemic, many case reports have described an association between the two conditions, and this review identified 23 papers that focused on COVID-19-related SAT. As outlined in **Table 1**, the common symptoms described by patients were neck pain and tenderness and symptoms of hyperthyroidism or thyrotoxicosis. More female patients were identified with SAT than male patients, which is not surprising as the incidence has been shown to be 19.1 vs. 4.1 per 100,000/year for women and men, respectively (64). The retrospective study by Batman et al. (18) stated the average age of patients was 41, with a mean onset time of 21 days (range: 5–39) from the time of a positive COVID-19 PCR result to the diagnosis of SAT. Similar findings were described in a recent review paper, where the mean age of patients was 40. However, the mean onset of symptoms was longer, with an average onset time of 4 weeks following SARS-CoV-2 infection (1). As there is an overlap of symptoms in SAT and COVID-19, such as fever, malaise, and lethargy, it can be difficult to distinguish which symptoms are due to SAT or COVID-19. This can lead to uncertainty and prolong the diagnosis of SAT, potentially delaying treatment and having detrimental effects on the prognosis of patients. Some of these symptoms were described in the case study by Henke et al. (21), where the patient experienced weakness and headaches that progressively worsened. However, the distinguishing symptoms were neck pain, palpitations, and hand tremors. These, coupled with laboratory investigations, allowed for the diagnosis of SAT. Furthermore, treatment of severe COVID-19 with high-dose corticosteroids can mask symptoms such as pyrexia and neck pain, making SAT harder to diagnose. Several studies identified the need for healthcare professionals to be aware of the relationship between SAT and COVID-19 infection.

The diagnosis of SAT is routinely based on laboratory tests and specific imaging. Laboratory results typically show a state of thyrotoxicosis, with negative TPO antibodies, as well as increased C-reactive protein (CRP) and erythrocyte sedimentation rate (65). Thyroid-specific imaging is used to further support the diagnosis of SAT. Thyroid ultrasound reveals a diffuse decrease in vascularity, heterogenous parenchyma, and hypoechoic areas in the thyroid gland, while thyroid scintigraphy demonstrates low or absent uptake of radioactive iodine within 24 h. An immune-mediated response is thought to occur after a viral infection, where cytotoxic T lymphocytes damage the thyroid follicular cells (1).

Over 50 years ago, the possibility of genetic influence increasing the susceptibility of developing SAT was first mentioned in the literature, exploring genotypes for human leukocyte antigen (HLA), which is involved in antigen presentation by T cells and antibody production by B cells in the immune system. Preliminary reports of a higher incidence of SAT in patients with the genotype HLA-B35 or HLA-B-67 have been recorded, with 70% of patients who develop SAT being carriers of HLA-B35 (66, 67). Other HLA haplotypes, such as HLA-B15/62 and HLA-Drw8, have also been implicated. One study suggested that viral insults like COVID-19 can trigger the stimulation of aberrant HLA DR isotype expression, as well as the activation of toll-like receptors (68).

The treatment of COVID-19-related SAT in all patients from studies examined in this systematic review involved NSAIDS and/or glucocorticoids, usually prednisolone, to reduce inflammation and eventually correct thyroid markers. Treatment of patients experiencing symptoms of hyperthyroidism, such as palpitations and tremors, may also involve drugs, such as beta blockers, to help alleviate these symptoms. On follow-up, one patient developed subclinical hypothyroidism, which was treated with levothyroxine (46). One patient also developed SAT alongside Graves’ disease (24), and another patient was diagnosed with an inflammatory nodule secondary to SAT but showed no signs of inflammation upon follow-up (53). These further complications highlight the need for long-term follow-up, even after SAT has been successfully treated.

The relationship between COVID-19 and SAT incidence was reported in one study, where the incidence of SAT was compared in patients with and without COVID-19. This was a population-based, retrospective, cross-sectional study that included 407,427 patients and found a positive correlation between COVID-19 patients and SAT diagnosis. Due to the large number of patients, the risk of bias was small, suggesting a possible association between COVID-19 infection and SAT (11).

### Graves’ disease post-COVID-19 infection

Graves’ disease is the most common form of hyperthyroidism, resulting from high levels of thyroid-stimulating immunoglobulins that activate the TSH-R. It is an autoimmune condition and commonly observed in middle-aged women. Two patients from the studies included in this review experienced thyroid storms, and one experienced Graves’ orbitopathy. Other rare symptoms identified include thyrotoxic periodic paralysis. Thyroid storm, in particular, is a life-threatening complication of Graves’ disease with a high level of mortality and requires timely detection and treatment in patients with clinical signs of thyrotoxicosis (66).

The population-based cohort study by Peng et al. (25) found a 1.3 hazard ratio (95% confidence interval: 1.10–1.54) between COVID-19 and Graves’ disease compared with COVID-19-negative patients, as well as an increased risk of Graves’ disease specifically in the 18–40-year-old age group with COVID-19. This suggest a possible link between COVID-19 and the development of Graves’ disease; however, more studies are needed to establish whether a correlation between COVID-19 infection and Graves’ disease is indeed present.

### Hashimoto’s thyroiditis after COVID-19

A few reports suggested that viral infections, including COVID-19, are able to trigger Hashimoto’s thyroiditis. This condition is a common autoimmune disease resulting from the infiltration of intrathyroidal mononuclear cells, which leads to the production of antithyroglobulin and antithyroid peroxidase antibodies, causing thyroid hormone derangement (29).

From the studies included in this review, only four patients were reported to develop Hashimoto’s thyroiditis after COVID-19 infection. These patients had overlapping symptoms at presentation, identical to those of patients with Hashimoto’s thyroiditis without COVID-19. Furthermore, treatment of Hashimoto’s thyroiditis involved replacement of thyroid hormones in the form of levothyroxine, which normalised the biochemical parameters and resolved clinical presentations of the patients (3).

### Painless thyroiditis after COVID-19

A number of reports described cases of COVID-19 patients who developed painless thyroiditis, which exhibits distinct characteristics compared to its counterpart, SAT. Painless thyroiditis, also known as silent thyroiditis, is thought to be a subtype of autoimmune thyroid disease or to a type of destructive thyroiditis (66).

A retrospective-prospective study focusing on patients who presented with SAT following COVID-19 infection described a total of 11 patients with COVID-19-associated thyroiditis, including five patients with painless thyroiditis. In this small series of patients, a comparison of symptoms allowed the following findings: symptoms consistent with painless thyroiditis presented earlier after COVID-19 infection than their comparator, and their levels of serum CRP and IL-6 were significantly higher (52). Patients with silent thyroiditis have also been reported to have a “transient” form of thyrotoxicosis, with high levels of serum thyroid hormones and low levels of TSH. This condition then spontaneously resolves, with the whole syndrome lasting for several months (66). Thyrotoxicosis is thought to be associated with an excessive inflammatory state that destroys thyroid follicles, thus causing the increase of thyroid hormones in the bloodstream, with elevated ESR (30).

The absence of anterior neck pain is thought to be related to the presence of lymphopenia associated with COVID-19. Within the thyroid gland, there is lower lymphocytic infiltration and giant cell formation, which in turn reduces the tension in the thyroid capsule and therefore does not cause any pain. These findings were also shown in the study by Mondal et al. (52), as patients with painless thyroiditis had low levels of absolute lymphocyte count and a high neutrophil-to-lymphocyte ratio. This study also found a “significant correlation” between IL-6 and free T4, total T4, and total T3, which could imply a role for proinflammatory cytokines in the development of painless thyroiditis (52).

The above studies also emphasised the need for increased awareness due to the “invisible” nature of this type of thyroiditis, especially in higher-risk patients, so that management can be delivered in a timely manner.

### Nonthyroidal illness syndrome after COVID-19

NTIS has been extensively reported in patients with COVID-19, although it is not exclusive to coronavirus infection, as it can occur during any type of severe illness, including myocardial infarctions, stroke, as well as during physiological stress and fasting. NTIS is considered an adaptive response to decrease energy expenditure during acute illness and, therefore, at least in the early stages of disease, to play a protective role. However, it has been linked to adverse effects and poor outcomes by prolonging recovery time, as the typical effect it exhibits on thyroid homeostasis lowers T3/fT3, with a consequential rise in reverse T3, but usually no effect on TSH and T4. The pathophysiology is complex, involving dysregulation of the HPT axis at multiple levels, including decreased peripheral conversion of T4 to T3 and decreased sensitivity of the pituitary TSH responses to decreased thyroid hormone levels (7, 69). In addition, prolonged secretion of cortisol following viral infection might be important, as it is thought to lower the activity of the hypothalamic–pituitary–adrenal (HPA) axis. The HPA axis can modulate the HPT axis, whereby acute stress can increase levels of TSH, whereas sustained stress can lower TSH release (3). Moreover, the role of inflammatory markers in NTIS has been investigated by numerous studies. Ilera et al. (70) found that thyroid hormone levels and their ratios (T3, T4, T3/T4, and fT3/FT4) negatively correlated with inflammatory markers such as CRP, LHD, fibrinogen and d-dimer. Inflammatory cytokines impairing the activity of deiodinase could provide a potential explanation (70). The severity of illness has been associated with the decrease in TSH, fT4, and fT3 due to the lower secretion levels of TRH. Therefore, the use of these thyroid levels has been investigated in relation to disease severity, with studies reporting a prognostic value for free T3. A number of studies showed that low fT3 levels had a significant association with disease severity and mortality (19); this was supported by another study, which showed that deceased patients due to COVID-19 had significantly lower T3 and TSH levels than survivors. The latter study also found that patients with the lowest T3 in their cohort (< 0.77 ng/ml) had higher rates of mechanical ventilation, intensive care unit admissions, and death when compared with patients with higher levels of T3 (> 1.00 ng/ml) (50). Additionally, the study by Baldelli et al. (13) found that hospitalised patients with COVID-19 had a “statistically significant reduction in fT3 and TSH” levels when compared to euthyroid controls, and patients admitted to the ICU had lower fT3 and TSH levels.

The frequency of COVID-19 patients developing NTIS was explored in a single-centre retrospective study by Arora et al. (19), where 58.8% of 102 patients exhibited NTIS. This was further supported in the retrospective study by Ahn et al. (50), which reported NTIS as the most common manifestation of thyroid dysfunction in a relatively small sample size of 119 patients. These findings highlight the need to monitor thyroid function and the possible development of NTIS in COVID-19 patients; enhanced surveillance of thyroid function can offer prognostic clues (7). In addition, a key recommendation in the review by Lui et al. (1) was that patients diagnosed with NTIS should be reassessed after 6 weeks of recovery from COVID-19, highlighting the need for monitoring, as around 15% of COVID-19 patients go on to develop thyroid abnormalities, with most cases being identified as NTIS. This, coupled with the potential prognostic implications of NTIS, could allow higher-risk patients to receive more timely intervention and improve their outcomes.

### Thyroid dysfunction in long COVID

Usually, patients with COVID-19 recover within up to 12 weeks; however, those who continue exhibiting varying symptoms after recovery have been termed as experiencing post-COVID-19 syndrome, post-acute COVID-19 syndrome, post-acute sequelae of SARS-CoV-2, and long COVID. As SARS-CoV-2 can affect the thyroid during the acute period, it is important to understand the impact on the thyroid during the post-acute infection period, associated with the development of chronic disease. Furthermore, data from reported cases of long COVID suggest that 53% share the symptom of fatigue, making it the most common symptom of the syndrome. This nonspecific symptom has led to the investigation of the relationship between thyroid function and long COVID (3).

A small observational study by Muller et al. (22) followed 75 COVID-19 survivors for 12 months, which included thyroid function assessment, ultrasound scans, and autoantibody assessment. They concluded that long-term thyroid consequences from COVID-19 “seemed unlikely”, as their results found that, at the end of their study period, all patients had normalised thyroid function and inflammatory markers, with no increased prevalence of autoantibodies. The only difference noted was the presence of focal hypoechoic areas in the thyroid gland, indicative of thyroiditis. This was seen for up to 1 year post-COVID-19 but was notably smaller in size when compared to those found during acute COVID-19 (22).

Conversely, a case-control study focusing on anosmia due to SARS-CoV-2 found a significant correlation between hypothyroidism and the prolongation of smell dysfunction in COVID-19 patients. It was postulated that this continuation of anosmia is due to direct virus-induced injury to the thyroid and olfactory nerve. Thyroid hormones regulate development of nearly all systems in the body, including the neural maturation of olfactory receptor neurons. Therefore, impaired thyroid hormone secretion or action due to SARS-CoV-2 could affect the development of these neurons, ultimately leading to COVID-19-induced anosmia (56). There are few studies focusing on potential thyroid dysfunctions during long COVID, highlighting the need for further research to understand the incidence and complications of long COVID and how the thyroid gland is related to this condition. However, the studies included in this review show an unclear pattern of the long-term effects of COVID-19 on the thyroid gland.

## Conclusion

Thyroid dysfunction is an endocrine complication frequently identified in the literature describing symptoms of COVID-19. Following SARS-CoV-2 infection, SAT, NTIS, and new-onset autoimmune thyroid disorders are the most common thyroid abnormalities. A number of studies included in this review also investigated the association between long COVID and thyroid disease, as well as autoimmune thyroid conditions. With the increased awareness of COVID-19-associated thyroid abnormalities, this could lead to improved detection of patient symptoms, especially those considered to be medically unexplained, such as chronic fatigue, by linking them to thyroid abnormalities. Furthermore, increasing evidence will eventually help to prevent complications, particularly in patients with multiple comorbidities, and reduce the risk of developing chronic conditions such as permanent hypothyroidism. Therefore, based on the evidence provided, thyroid function should be considered in patients displaying relevant clinical features so that management of these patients can be tailored and comprehensive care offered in a timely manner.

However, it is important to acknowledge the limitations of our study. Conflicting results identify the need for additional high-quality studies with larger, well-characterised patient groups. Furthermore, the majority of studies were case reports, where causality cannot be inferred due to the lack of control groups. However, the increase in case reports has warranted further research into thyroid dysfunction following COVID-19 infection, as demonstrated through the retrospective and prospective studies included. Nevertheless, as our study is a systematic review, it has inherent strengths, such as transparent and reproducible stages in our methodology, ensuring a low risk of bias, as results are generated based on the defined PICO criteria, which are clearly stated in **Table 2**.

## Data Availability

The original contributions presented in the study are included in the article/**Supplementary Material**. Further inquiries can be directed to the corresponding author.
