# Supplementary material for: Thyroid function during COVID-19 and post-COVID complications in adults: a systematic review
Source: Front Endocrinol (Lausanne). 2025 Feb 4;15:1477389. doi: 10.3389/fendo.2024.1477389 (PMC11832367; doi:10.3389/fendo.2024.1477389)
Supplement: Supplementary file 1 [file Table1.docx]

**Supplementary Table 1** The details of the included studies in this systematic review.

| Author and publication year | Title | Country | Study design | Number of patients | Patient data: age, gender, severity of COVID-19 | Thyroid characteristics post COVID infection |
| --- | --- | --- | --- | --- | --- | --- |
| R. Baldelli  (Baldelli et al., 2021) | Thyroid dysfunction in COVID‐19 patients | Italy | Retrospective study | 46 | Age = 31-35 years  Gender = 14 females, 32 males  Severity = 23 patients were moderate, and 23 patients were severe | - 28 patients had low serum fT3 levels. - All patients had low serum TSH levels. - Patients were diagnosed with NTIS. - Some patients in intensive care had already received high doses of glucocorticoids which can lower TSH and fT3. |
| Álvarez Martín MC  (Alvarez Martin et al., 2021) | Subacute De Quervain thyroiditis after SARS-CoV-2 infection | Spain | Case study | 1 | Age = 46  Gender = female  Severity = unknown | - Neck pain, worse on swallowing - Low grade fever - Malaise - Insomnia - Grade 1 goitre - Diagnosed with subacute thyroiditis |
| Maham A. Mehmood (Mehmood et al., 2020) | A Case of Post-COVID-19 Subacute Thyroiditis | USA | Case study | 1 | Age = 29  Gender = female  Severity = unknown | - Fever - Odynophagia - Exertional tachycardia - Shortness of breath - Diagnosed with subacute thyroiditis |
| Alessandro Brancatella  (Brancatella et al., 2023) | Subacute Thyroiditis After Sars-COV-2 Infection | Italy | Case study | 1 | Age = 18  Gender = female  Severity = mild | - Fever - Fatigue - Palpitations - Anterior neck pain, radiating to jaw. - Thyroid ultrasound showed multiple, diffuse hypoechoic areas. - Subacute thyroiditis was diagnosed |
| E. Asfuroglu Kalkan  (Asfuroglu Kalkan and Ates, 2020) | A case of subacute thyroiditis associated with Covid‐19 infection | Turkey | Case study | 1 | Age = 41  Gender = female  Severity = unknown | - Fever - Neck pain - ‘Thyroid ultrasound examination showed a relative diffuse decrease of vascularity and parenchyma was heterogeneous. - Subacute thyroiditis was diagnosed |
| Adnan Batman  (Batman et al., 2023) | Subacute Thyroiditis Related to SARS-CoV-2 Vaccine and Covid-19 (THYROVAC Study)  : A Multicentre Nationwide Study | Turkey | Nationwide, multicentre, retrospective cohort study | Total = 811  Patients with subacute thyroiditis related to COVID19 = 98 | Age = 36-50  Gender = 34 males, 64 females  Severity = mild to moderate | - ‘No statistical difference was found between the SAT aetiology groups in terms of age, sex, thyroid disease in the past, smoking, and familial thyroid disease history. - the time between PCR positivity and diagnosis in the Cov-SAT group was 21 days (range, 5-39 days)’ - most common symptoms were neck pain and tenderness |
| Saurabh Arora (Arora et al., 2022) | Endocrine and Metabolic Manifestations of COVID-19 Patients Admitted to an Intensive Care Unit | India | single-centre, retrospective study | 102 | Age = 55.5 ± 14.8  Male to female ratio = 3.25:1  Severity = moderate (18), severe (60) and critical (24) | - 58.8% of patients had NTIS. - 4.9% of patients had thyrotoxicosis which were later diagnosed with SAT. - 1.9% of patients had overt hypothyroidism. - All patients demonstrated decreased to absent uptake on thyroid scintigraphy. - ‘Logistic regression analysis demonstrated that low FT3 levels showed a significant association with increased severity and mortality.’ |
| Katrin Henke  (Henke et al., 2023, Nham et al., 2023) | Subacute Thyroiditis Complicating COVID-19 Infection | Switzerland | Case study | 1 | Age = 41  Gender = female  Severity = mild | - Progressive symptoms of neck pain, headache, asthenia, insomnia, hands tremor, hyperhidrosis, diarrhoea, weight loss of 8kg, and palpitations. - ‘Thyroid ultrasonography showed an enlarged thyroid gland of up to 50 ml with an inhomogeneous, predominantly hypoechogenic structure, and mild hyperperfusion over the entire organ.’ - Diagnosed with SAT |
| Jeongmin Lee (Lee, 2023) | Beyond Acute COVID-19: Investigating the Incidence of Subacute Thyroiditis in Long COVID-19 in Korea | Korea | population-based retrospective and cross-sectional study | 407,427 | Age = 46.3±17.4  Gender = 209,897 – male, 197,530 – female.  Severity = unknown | - The events of SAT were 87.6% in women and 12.4% in men. - ‘In COVID-19 patients, 61 cases of SAT were reported during 366,346 person-years of follow-up, resulting in a higher incidence rate than non-covid patients.’ |
| Ilaria Muller  (Muller et al., 2023) | Long-term outcome of thyroid abnormalities in patients with severe Covid-19 | Italy | single-centre observational study | 183 | Age = mean (±s.d.) age of 67.5 ± 13.8 years  Gender = males (*n* = 124, 68%)  Severity = severe | - ‘Thyrotoxicosis was observed in 12/120 (10%) patients (mean age 68 ± 11 years, 9 males and 3 females) untreated with steroids at hospital admission; none complained of neck pain and were therefore diagnosed with atypical thyroiditis.’ - ‘Hypothyroidism was observed in 7/120 (6%) patients (mean age 78 ± 12 years, 3 males and 4 females).’ |
| A.S. Franca (Franca et al., 2023) | Graves’ disease: A new association with COVID-19? | Spain | Clinical letter with a case study | 1 | Age = 30  Gender = female  Severity = mild | - Tachycardia - Palpitations - Fatigue - Dizziness - Tremors - Excessive sweating - Goitre and the ultrasound showed an enlarged thyroid, homogeneous, without nodular images. - Low TSH and high T3 and T4 levels in serum. - Diagnosed with graves disease. |
| Eliel Nham  (Nham et al., 2023) | Concurrent Subacute Thyroiditis and Graves’ Disease After COVID-19: A Case Report | Korea | Case study | 1 | Age = 27  Gender = female  Severity = mild | - Chest pain worse on expiration - Fever - Neck stiffness - FT3 was high while TSH was low indicating thyrotoxicosis. - ‘Computed tomography (Okwor et al.) of the neck revealed mild enhancement and swelling of the bilateral palatine tonsils, bilateral cervical lymph node enhancement, and mild heterogeneous enhancement of both thyroid glands.’ - ‘Ultrasonography of the thyroid gland showed architectural distortion and increased blood flow in the bilateral thyroid glands, consistent with thyroiditis.’ - ‘fluctuation of TFTs—from overt thyrotoxicosis to a subclinical state and again to overt thyrotoxicosis in the short term of 2 months and a high TSI confirmed the diagnosis of Graves disease.’ |
| Kuan Peng  (Peng et al., 2023) | Risk of autoimmune diseases following COVID-19 and the  potential protective effect from vaccination: a population-  based cohort study | China | population-  based cohort study | 1,028,721 | Age = mean age – 56.68  Sex = 44.6% male, 55.4% female  Severity = unknown | - Graves’ disease presented an increased risk only among the 18–40-year-old age group. - There was a 0.78 hazard ratio of incident Hashimoto’s thyroiditis. - There was a 1.3 hazard ratio of incident graves disease. |
| Angela Shermetaro (Shermetaro and Bushman, 2023) | New-Onset Graves’ Disease With Thyroid Storm After COVID-19 Infection | USA | Case report | 1 | Age = 81  Gender = male  Severity = mild | - Persistent dizziness and weakness - Tremors - Eye irritation - Voice hoarseness - Headaches - Fogginess - The patient was in AF with rapid ventricular response and admitted to palpitations. - Palpable thyroid - ‘The patient had a Burch-Wartofsky score of 45, and the clinical diagnosis of thyroid storm was made.’ - His thyroid technetium scan demonstrated homogenous uptake compatible with Graves’ disease. |
| Dennis C. Boyle (Boyle and Mullally, 2023) | Thyrotoxicosis after COVID-19 Infection with a Delay in Graves’ Disease Antibody Positivity | USA | Case report | 1 | Age = 65  Gender = female  Severity = mild | - Progressive exertional dyspnoea - Palpitations - ‘Thyroid ultrasound showed a homogenous gland without asymmetry, nodules or focal lesions, and normal vascularity on colour flow Doppler.’ - ‘With a very low TSH and signs of severe thyrotoxicosis with the potential for impending thyroid storm (Burch Wartofsky score = 35).’ - Undetectable TSH with high total T4, free T4, and total T3 from her bloods. |
| Zheng Cong Lee (Lee et al., 2023) | Hashimoto’s thyroiditis-related myopathy in a patient with SARS-CoV-2 infection | Singapore | Case report and systematic literature review | 1 | Age = 33  Gender = male  Severity = mild | - ‘The patient’s free T4 level was lower than the reference range, with a value of less than 5.50 pmol/L (normal: 10.00–20.00 pmol/L). - Thyroid- stimulating hormone (TSH) levels were markedly increased to 216 mIU/L, well above the reference range of 0.400 to 4.00 mIU/L. - The patient’s anti-TPO levels were elevated to 78.53 IU/ mL, compared to the normal value of less than 5.50 IU/mL. - Evaluation of these results led to HT and probable hypothyroidism-related myopathy.’ |
| Alessandro Rossini  (Rossini et al., 2023) | Increased prevalence of autoimmune thyroid disease after COVID-19: A single-centre, prospective study | Italy | Single centre, prospective study | 494 | Age = 55-73  Gender = 132 females and 362 males  Severity.= mild to severe | - Ultrasonographic features of thyroiditis were present in 94.9% of the evaluated patients with positive antibodies. - TSH was within the normal range in 95% of patients. - ‘Autoimmune thyroid disease prevalence in COVID-19 survivors was doubled as compared to age and sex-matched controls.’ |
| Karen Feghali (Feghali et al., 2021) | Manifestations of thyroid disease post COVID-19 illness: Report of Hashimoto thyroiditis, Graves’ disease, and subacute thyroiditis | USA | Case report | 3 | Age = patient 1 – 38, patient 2 – 33, patient 3 - 41  Gender = all female  Severity = all mild | - ‘Patient 1 diagnosed with Hashimoto’s thyroiditis and hypothyroidism   - anterior neck discomfort and noticed thyroid enlargement along with extreme fatigue, dry skin, hair loss, and worsening depression.   - TSH 136 mIU/L (range 0.34–5.6), free T4 level 0.2 ng/dL (range 0.93–1.7), anti-thyroid peroxidase antibody >900 IU/mL (normal less than 9) and anti-thyroglobulin antibodies >1000 IU/ml (normal less than 1).   - Thyroid ultrasound indicated thyromegaly with a heterogenous and hypoechoic sonographic appearance.   - A fine needle aspiration biopsy targeting a diffusely heterogeneous and hypoechoic part of the left upper pole indicated the presence of a small number of follicular cells along with mixed inflammatory cells, including groups of histiocytes with epithelioid morphology suggestive of granulomatous inflammation, without clear lymphocytic infiltration. - Patient 2 diagnosed with graves’ disease   - palpitations and shortness of breath accompanied by worsening fatigue.   - TSH <0.01 mIU/L (range 0.4–4.5), Free T4 2.1 ng/dl (range 0.8–1.8), total T3 216 ng/dl (range 76–181), elevated TSI 309 (normal <140%), thyroglobulin normal 8.8 ng/ml (range 2.8–40.9), CRP and ESR normal.   - The thyroid ultrasound showed mild thyromegaly with heterogeneous and diffusely hypervascular sonographic appearance. A 24-h thyroid uptake was calculated at 47.1% (normal values between 8% and 35). - Patient 3 diagnosed with subacute thyroiditis   - persistent palpitations and insomnia   - TSH 0.01 mIU/L, free T4 1.9 ng/dL and positive thyroid peroxidase anti- bodies 69 IU/mL (normal less than 9).   - A nuclear medicine thyroid up- take and scan indicated an abnormal 24-h thyroid radioiodine uptake, calculated at 0.09%, consistent with a diagnostic of thyroiditis.’ |
| Rafael Silvestre Knack (Knack et al., 2021) | Hashimoto’s thyroiditis following SARS- CoV-2 infection | Brazil | Case report | 1 | Age = 33  Gender = female  Severity = mild | - Symptoms of fatigue and severe hair loss - ‘TSH levels of 8 mIU/mL (upper limit of the normal range of 4.3 mIU/mL); free T4 of 0.5ng/dL (normal range 0.7–1.8ng/dL) and antibodies, anti-Tg of 252 IU/mL and anti-TOP of 115IU/mL (normal range less than 60IU/mL for both). - Ultrasonographic examination of the thyroid showed the presence of diffusely hypoechoic and heterogeneous glands. - the patient performed a genetic test, which assesses the genotyping of markers, observing a predisposition to exacerbation of proinflammatory cytokines, supported by polymorphisms in the tumour necrosis factor (TNF) alpha and interleukin (IL)-6 genes.’ |
| David Tak Wai Lui  (Lui, 2021) | Insights from a Prospective Follow-up of Thyroid Function and Autoimmunity among COVID-19 Survivors | China | Prospective study | 122 | Age = 44 to 63  Gender = 60 male patients and 62 female patients  Severity = 99 mild patients, 19 moderate patients and 4 severe patiens | - 2% of patients had new onset abnormal TFTs on admission. - ‘85.2% patients had anti-thyroid antibody titres reassessed which had an increase in anti-TPO titre upon reassessment. - However, 70% of patients received interferon beta-1b, potentially confounding the analysis of SARS-CoV-2-related incident thyroid dysfunction and autoimmunity.’ - Most of the patients did not have thyroid imaging. |
| Rebeca Barahona San Millán (Barahona San Millan et al., 2020) | Painless thyroiditis in SARS-CoV-2 infection | Spain | Case study with a letter to the editor | 1 | Age = 52  Gender = male  Severity = Moderate | - Laboratory testing revealed, elevated thyroxine, normal triiodothyronine, supressed thyrotropin and negative autoimmunity. - ‘Thyroid scintigraphy was performed; this showed increased vascular fundus uptake with virtually no thyroid gland activity, suggestive of thyroiditis.’ - The patient had no symptoms of hyperthyroidism. |
| Tsering Dolkar  (Dolkar et al., 2022) | Painless Subacute Thyroiditis in a Patient with Acute COVID-19 Infection: A Transient Event | USA | Case study | 1 | Age = 55  Gender = female  Severity = severe | - This patient was diagnosed with painless subacute thyroiditis due to the absence of pain and abnormal thyroid markers. - On day 1 her TSH was 0.075 uIU/mL (Piekarska et al.), FT4 was 2.51 ng/dL (high), and T3 was 99 ng/dL. |
| Bruno Sousa (Sousa et al., 2022) | Graves’ Disease Caused by SARS-CoV-2 Infection | Portugal | Case report | 1 | Age = 28  Gender = male  Severity = mild | - 3-week history of fatigue on medium exertion, shortness of breath and palpitations. - The patient experienced significant unexplained weight loss of 8 kg during this time, despite a normal appetite. - Examination showed non-tender, symmetrical, enlargement of the thyroid gland. - TSH level of <0.001 mU/ml, free T4 of 7.11 ng/dl, and total T3 of 486 ng/dl. Autoimmunity work-up revealed elevated TRAbs at 8.1 IU/l (positive >1.5 IU/l), anti-peroxidase antibody at 478 IU/ml (<34 IU/ml) and anti-thyroglobulin antibodies at 352 IU/ml (<115 IU/ml). The thyroid values from the previous year were normal. - Thyroid ultrasound was performed and showed a diffusely heterogeneous and irregular thyroid, and a nodular image below the sternal notch. To confirm this nodular image, thyroid scintigraphy was performed, which excluded the nodule and confirmed a Graves’ disease pattern.’ |
| A.M. Urbanovych,  (Urbanovych et al., 2021) | Coronavirus as a Trigger Of Graves’ Disease | Ukraine | Case report | 1 | Age = 22  Gender = female  Severity = moderate | - Palpitations, hands tremor, muscle weakness, anxiety and sleep disturbance. - Fever and general weakness. - ‘TSH <0.010mU/L (normal values 0.4- 4.0mU/L); FT3 was 12.55 pg/mL (n. v. 2.0–4.4); and FT4 was 17.77 ng/dL (n. v. 0.93–1.77). Thyroglobulin (Tg) was 421.5 pg/mL; (n.v. 3.5-77.0), whereas TRAbs were extremely increased - 32.72 mU/mL (n.v.<1.58 mU/mL). ‘ - Neck ultrasonography showed a diffusely enlarged, hypoechogenic thyroid gland with markedly enhanced vascularization. |
| Asaf Harris (Harris and Al Mushref, 2021), MD | Graves’ Thyrotoxicosis Following SARS-CoV-2 Infection | USA | Case report | 1 | Age = 21  Gender = female  Severity = mild | - 3 days of progressively worsening tachycardia with palpitations, anxiety, and shortness of breath. - ‘Initial thyroid tests revealed a TSH level of 0.01 (0.30-5.00) mcIU/mL with a free thyroxine (T4) level of 3.8 (0.6-1.6) ng/dL - At follow up her laboratory results showed TSH, free T4, free triiodothyronine, thyroid-stimulating immunoglobulin, and thyrotropin receptor antibody levels of <0.01 (0.30-5.00) mcIU/ mL, 3.6 (0.6-1.6) ng/dL, 15.2 (2.2-3.9) pg/mL, 2.6 (<1.3) thyroid- stimulating immunoglobulin index, and 17 (0.00-1.75 IU/L) IU/L, respectively.’ |
| Dimitra Argyro Vassiliadi  (Vassiliadi et al., 2021) | Thyroid hormone alterations in critically and non-critically ill patients with SARS-CoV-2 infection | Greece | Cohort observational study | 102 | Age = mean age 59.3 ± 18.3 years  Gender = 66.3% male  Severity = 15 – mild, 46 – moderate and 41 – severe | - T3 and TSH levels were lower in the ICU patients, compared to ward patients but there was no difference between patients with or without COVID-19. - FT4 levels were similar between ICU and ward patients - ‘The prevalence of hypotriiodothyroninemia (low T3) levels was significant higher in ICU patients (70.0%) compared to ward patients (45.5%) and outpatients (6.7%), - Thyroid hormone abnormalities consistent with either overt or subclinical thyrotoxicosis were seen in 14.6% in the ICU patients and 4.4% of the ward patients. - Overall, both overt and subclinical thyrotoxicosis was observed in 8.8% of all SARS-CoV-2 positive patients and 7.4% of the SARS-CoV2 negative patients.’ |
| Magdalena Swistek  (Swistek et al., 2022) | Euthyroid Sick Syndrome as a Prognostic Indicator of COVID-19 Pulmonary Involvement, Associated With Poorer Disease Prognosis and Increased Mortality | Poland | Retrospective study | 215 | Age = 58 to 78  Gender = 93 female and 122 male  Severity = mild, moderate and severe | - 82 patients with COVID-19 had euthyroid sick syndrome. - ‘Patients with ESS were older than the ones without ESS: 73 years (IQR, 66.0; 82.3) vs 65 years (IQR, 52.5; 74.0) (P < .001). - Patients with ESS had a significantly higher mortality rate during hospitalization than those without, 28 out of 82 (34.1%) for those with ESS vs 15 out of 133 (11.3%) for those without - The number of patients requiring high flow nasal oxygen therapy for ESS was 7 out of 82 (8.5%) vs 3 out of 133 (2.3%) for non-ES - The number of patients intubated and transferred to the ICU for ESS was 15 out of 82 (18.3%) vs 8 out of 133 (6.0%) for non ESS.’ |
| C.Sparano (Sparano et al., 2022) | Euthyroid Sick syndrome as an early surrogate marker of poor outcome in mild SARS-CoV-2 disease | Italy | Prospective study | 506 | Age = 68.8 ± 15.6  Gender = 315 male and 191 female  Severity = mild | - ‘The most frequent thyroid disorder was ESS (57%). Admission levels of fT3 were significantly lower within the unfavourable outcome subgroup and were negatively associated with several poor prognostic markers, including IL-6. - In Kaplan-Meier and Cox regression analyses, fT3 was independently associated with poor outcome and death. - In the lower FT3 subgroup there was a 3.5-fold increased risk of negative outcomes and shorter survival.’ |
| Chika Juliet Okwor  (Okwor et al., 2021) | Assessment of thyroid function of newly diagnosed SARS-CoV-2 infected patients in Nigeria | Nigeria | Cross-sectional study | 90 (45 COVID-19 patients and 45 control patients) | Age = mean age of COVID-19 patients 35.31±12.44 years  Gender = from the 45 COVID-19 patients, 34 were male and 11 were female  Severity = unknown | - The mean plasma TSH and FT3 concentrations were significantly higher in COVID- 19 patients compared to controls. - Amongst the cases were 7 (15.6) sick euthyroid and 3 (6.7%) subclinical hypothyroidism whereas none was found among the controls. |
| Jing Gong (Gong et al., 2021) | Prognostic significance of low TSH concentration in patients with COVID-19 presenting with non-thyroidal illness syndrome | China | Retrospective cohort study | 150 | Age = median age was 69.5 years  Gender = 69 female and 81 male  Severity = 66 patients (44%) were critically ill, 59 patients (39.33%) were seriously ill, 25 patients (16.67%) were mildly ill, and 46 patients (30.67%) died. | - 68% of patients included had normal FT4 and TSH levels. - Critical illness rates were significantly higher in the low TSH group than in the normal TSH group. - The mortality rate was significantly higher in the low FT4 group. - Low TSH levels were independently related to 90-day mortality. |
| Yair Schwarz (Schwarz et al., 2021) | Sick Euthyroid Syndrome on Presentation of Patients With COVID-19: A Potential Marker for Disease Severity | Israel | Retrospective cohort study | 54 | Age = 48-68  Gender = -  Severity = mild to severe | - Patients with the lowest FT3 had significantly higher mortality, mechanical ventilation and intensive care unit admission. - FT3 was found to be a significant independent predictor of mortality. |
| Eugenia Campos-Barrera (Campos-Barrera et al., 2020) | Subacute Thyroiditis Associated with COVID-19 | Mexico | Case report | 1 | Age = 37  Gender = female  Severity = mild | - ‘Severe neck pain (8/10) irradiating to the right jaw and ear as well as fatigue. - Physical exam showed moderately enlarged tender thyroid gland and neck adenopathies. - The thyroid tests were positive for hyperthyroidism with an undetectable TSH, T4 total 13.5 mcg/dL, T4 free 1.6 ng/dL, and T3 total 211 ng/dL. - Thyroid iodine scan showed no radioactive iodine uptake and subacute thyroiditis was confirmed. ‘ |
| Saeed Sohrabpour (Sohrabpour et al., 2021) | Subacute Thyroiditis in COVID-19 Patients | Iran | Case reports | 6 | Age = P1 – 26, P2- 37, P3 – 35, P4 – 41, P5 – 52 and P6 – 34  Gender = P1 – female, P2- female , P3 – male, P4 – female, P5 – male and P6 – female  Severity = all patients were mild | - ‘All 6 patients presented to hospital with fever, palpitations and anterior neck pain. - Physical examination revealed painful, tender, and slightly enlarged thyroid gland. - The ultrasonographies showed bilateral hypoechoic areas in thyroid gland which was suggestive of SAT. - Laboratory results showed mildly elevated free thyroxine and free triiodothyronine with undetectable thyrotropin. - C-reactive protein levels were also slightly elevated, and in all patients, erythrocyte sedimentation rate was more than 60 mm/h.’ |
| Akshay Khatri (Khatri et al., 2021) | Subacute Thyroiditis from COVID-19 Infection: A Case Report and Review of Literature | USA | Case report and literature review | 1 | Age = 41  Gender = female  Severity = mild | - ‘Worsening pain and swelling of her anterior neck for 6 weeks and persistent fevers for 3 weeks. - The pain radiated to her jaw and was associated with odynophagia. - Followed by fever, chills and diaphoresis. - She noted 6 kg unintentional weight loss, fatigue, alopecia, heat intolerance, irritability, head- aches, bilateral hand tremors, and palpitations. - Thyroid function tests (TFTs) revealed a low thyroid-stimulat- ing hormone (TSH) with normal T3 uptake. - Repeat TFTs showed low TSH and elevated thyroid hormone levels, suggestive of thyrotoxicosis. - A thyroid ultrasound revealed a heterogenous thyroid gland (right lobe: 4.3 × 2.3 × 1.6 cm; left lobe 4.3 × 1.9 × 1.4 cm) with bilateral patchy ill-defined hypoechoic areas, suggestive of subacute thyroiditis.’ |
| Adrian Whiting (Whiting et al., 2021) | Post-COVID-19 Fatigue: A Case of Infectious Hypothyroidism | USA | Case report | 1 | Age = 49  Gender = Male  Severity = mild | - Six-month history of fatigue, unintentional 10-pound weight gain, constipation, dry skin, and myalgia. - His fatigue was noticeable to the patient and severely affected his activities of daily living. - Lab results were significant for elevated thyroid-stimulating hormone (TSH) of 74 and a detectable anti- thyroid peroxidase level of 626. - Diagnosis of hypothyroidism was made. |
| L. Davoodi  (Davoodi et al., 2021) | A 33-year-old man with COVID-19 presented with subacute thyroiditis: A rare case report and literature review | Iran | Case report | 1 | Age = 33  Gender = Male  Severity = unknown | - ‘Presented to the clinic with fever (38.5°C), sore throat, body aches and lethargy for 2 days. - Six days later he presented to the Emergency Room with fever (39°C) and chills, sweating, sore throat and dry cough. - On examination slight tenderness in the neck in the thyroid area - On thyroid ultrasound, a heterogeneous thyroid gland with bilateral ill-defined hypoechoic areas revealed SAT. - His thyroid function tests were thyroid-stimulating hormone (TSH) < 0.001 mUI/L, total thyroxine 23.1 μg/dL (normal range 4–11 μg/dL) and total triiodothyronine 236 ng/dL.’ - Thyroperoxidase antibody and thyrotropin receptor antibody were negative. |
| Rosaria Maddalena Ruggeri (Ruggeri et al., 2021) | Subacute thyroiditis in a patient infected with SARS-COV-2: an endocrine complication linked to the COVID-19 pandemic | Italy | Case report | 1 | Age = 43  Gender = female  Severity = mild | - ‘The patient suddenly developed pain and tender- ness in the anterior cervical region, fatigue, tremors, and palpitations. The neck pain, which was severe and was aggravated by swallowing. - Thyrotropin (TSH) was suppressed (0.006 mU/L; normal values 0.27–4.2); free triiodothyronine (FT3) was 7.03 pg/mL (n. v. 1.71–3.71); and free thyroxine (FT4) was 2.69 ng/dL (n. v. 0.7–1.48). Thyroglobulin (Tg) was markedly increased (188 pg/mL; n.v. 0–40), whereas anti-thyroglobulin antibodies (TgAb), anti-peroxidase anti- bodies (TPOAb), and anti-TSH receptor antibodies (TRAb) were undetectable. - Physical examination revealed mild tremors of the extremities, a diffuse and painful goiter, and enlarged and tender cervical and submandibular lymph nodes. - Neck ultrasonography showed a diffusely enlarged and hypoechogenic thyroid gland. Thyroid scintigraphy showed markedly reduced 99mTc-perthecnetate uptake in the gland. - SAT was diagnosed.’ |
| Alessandro Brancatella (Brancatella et al., 2020a) | Is subacute thyroiditis an underestimated manifestation of SARS-CoV-2 infection? Insights from a case series | Italy | Case reports | 4 | Age = 29-46  Gender = female  Severity = mild | - ‘Neck pain radiated to the jaw and palpitations were the main presenting symptoms and were associated with fever and asthenia. - Thyroid function tests (available in three subjects) were suggestive of destructive thyroiditis and inflammatory markers were high. At neck ultrasound the thyroid was enlarged, with diffuse and bilateral hypoechoic areas and (in three patients) absent vascularization at colour doppler.’ |
| Szofia Hajósi-Kalcakosz (Hajosi-Kalcakosz et al., 2022) | Subacute thyroiditis associated with COVID-19 infection: a report of an increasing entity | Hungary | Case report and review | 1 | Age = 31  Gender = female  Severity = mild | - ‘Developed severe neck pain and tenderness that radiated to the jaw, and a few days later, she experienced fever (38.7 oC), fatigue, myalgia, palpitation, and tremor. - Laboratory test showed high levels of both FT4 1.60 ng/dL (normal range: 0.70- 1.48 ng/dL) and FT3 4.10 pg/mL (normal range: 1.71- 3.71 pg/mL), low level of TSH 0.046 uIU/mL (normal range: 0.350-4.940 uIU/mL). - On neck ultrasound, both thyroid lobes had normal size with normal vascularization. The left lobe was diffuse hypoechoic (Figure 1), and in the right lobe, a 15 × 8 × 7 mm hypoechoic area was detected with slurred border.’ - SAT was confirmed. |
| Sameh Samir Elawady  (Elawady et al., 2022) | A Case of Subacute Thyroiditis following COVID-19 Infection | Nepal | Case report | 1 | Age = 33  Gender = female  Severity = mild | - Four weeks of anterior neck pain as well as swelling - The patient also had also had fever, malaise, and myalgia. - ‘Laboratory investigations showed low TSH level (0.04mU/L), normal free T4, normal free T3, negative thyroid peroxidase antibody (TPO) (0.9 IU/mL), and negative thyroid-stimulating immunoglobulin (Vassiliadi et al.) (1.0). - Ultrasound of the neck revealed a heterogeneously enlarged thyroid gland with two small 4mm solid hypo- echoic solid nodules in the isthmus.’ |
| Eirik Tjønnfjord  (Tjonnfjord et al., 2021) | Subacute thyroiditis after COVID-19 | Oslo | Case report | 1 | Age = 40’s  Gender = male  Severity = mild | - ‘Symptoms included fever (38.3 °C), dysphagia, headache, a mild dry cough, dyspnoea and general myalgia. - Diffuse tenderness to palpation was noted on both sides of the throat, but there was no enlargement of the thyroid. - Thyroid-stimulating hormone (TSH) 0.01 mIU/L (0.35–3.6), FT4 (tetraiodothyronine) 27.8 pmol/L (9–19), FT3 (triiodothyronine) 7.5 pmol/L (2.6–5.7), thyroperoxidase (P-TPO) <3 (<6) and TSH receptor antibodies (TRAb) <0.9 (<1.8). - An ultrasound of the neck showed slight diffuse enlargement of the thyroid gland with discrete surrounding oedema and heterogeneous parenchyma, consistent with thyroiditis.’ |
| Woon H. Chong (Chong et al., 2021) | Subacute Thyroiditis in the Setting of Coronavirus Disease 2019 | USA | Letter to the editor with a case report | 1 | Age = 37  Gender = male  Severity = mild | - ‘Physical examination of the neck revealed a non-enlarged thyroid gland that is diffusely tender to palpation without any lymphadenopathy. - The thyroid gland ultrasound scan that revealed diffusely heterogeneous echotexture. - Free thyroxine (T4) and total triiodothyronine (T3) levels were elevated at 2.3 ng/L and 202 ng/L, respectively. Thyroid-stimulating hormone (TSH) was low at 0.01 mU/L. Thyroid peroxidase antibody (TPO Ab.), thyroid-stimulating immunoglobulin (Vassiliadi et al.) was negative. - The patient returned three weeks later with new reports of fatigue, anorexia, weight gain, constipation, and puffiness of his face. - Repeat laboratory workup revealed a paradoxical increase in TSH of 15 mU/L, but free low T4 and total T3 at 0.1 ng/L and 10 ng/L, respectively and the diagnosis of hypothyroidism was made.’ |
| Daisuke Sato (Sato et al., 2021) | Successful Management of Subacute Thyroiditis Following SARS-CoV-2 Infection | Japan | Case report | 1 | Age = 31  Gender = female  Severity = mild | - Persistent low-grade fever - ‘Physical examination revealed that the left side of the thyroid gland was painful, hard, and slightly enlarged. The pain had spread to the right lobe. Her neck pain had radiated to the jaw. - Laboratory test results showed undetectable serum thy- rotropin (TSH) levels (0.0 μIU/mL; normal ranges, 0.4-4.0); free thyroxine (FT4) levels of 3.2 ng/dL (normal ranges, 0.8-1.9); and free triiodothyronine (FT3) levels of 7.3 pg/mL (normal ranges, 2.2-4.1). - Thyroid peroxidase antibodies and TSH receptor antibodies were negative. The patient tested positive for the thyroglobulin antibody. - Thyroid ultrasound showed diffuse hypoechoic areas consistent with the pain. - Thyroid scintigraphy showed no remarkable uptake of technetium-99m within the left lobe of the thyroid gland. - Diagnosis of SAT was made.’ |
| Mohamed Sadoon Al‐Shammaa (Al-Shammaa and Abdlkadir, 2022) | A case of post COVID‐19 subacute thyroiditis | Iraq | Case report | 1 | Age = 53  Gender = female  Severity = mild | - ‘On examination the patient had a sore and soft neck, an increased pulse (134 bpm), and tremors. - T3, T4 was raised and TSH was decreased. - Anti-thyroperoxidase and Anti‐thyroglobulin antibody was increased. - ^99m^Tc‐thyroid scan and uptake showed no uptake at thyroid gland indicative of subacute thyroiditis.’ |
| Deniz Gezer (Gezer and Ecin, 2022) | EFFECTS OF COVID-19 INFECTION ON THYROID FUNCTIONS | Turkey | Retrospective study | 201 | Age = mean age of 51.9 ± 14.6 years  Gender = 121 males, 80 females  Severity = 44 mild, 129 moderate and 28 severe | - ‘110 patients had low TSH and diagnosed with subclinical hyperthyroidism. - 116 patients had low T3, 42 had low T4, 3 had high T4 and 33 had anti-TPO elevation. - Increased length of hospital stays (p=0.04), clinical severity (p=0.05), high LDH level (p= 0.008) and high D-dimer (p=0.02) were found significant in patients with low TSH. - Low TSH was detected more frequently in moderate patients.’ |
| Jiyeon Ahn (Ahn et al., 2021) | Thyroid Hormone Profile and Its Prognostic Impact on the Coronavirus Disease 2019 in Korean Patients | Korea | Retrospective study | 119 | Age = mean 64.3±16.8  Gender = 62 male, 57 female  Severity = 32 mild-moderate and 87 severe-critical | - ‘NTIS was the most common manifestation (18.5%) among patients with abnormal thyroid function parameters, followed by subclinical thyrotoxicosis (14.3%). - Patients with more severe COVID-19 infection had lower TSH and T3 levels. - TSH and T3 levels were significantly lower in non-survivors than in survivors. - 81.3% of the patients with non-severe COVID-19 were euthyroid, whereas 57.5% of the patients with severe to critical disease and 35.3% of deceased patients were euthyroid at COVID-19 presentation. - Lowest T3 level (<0.77 ng/mL) was independently associated with COVID-19-related mortality.’ |
| Khary Edwards (Edwards and Hussain, 2021) | Two Cases of Severe Autoimmune Thyrotoxicosis Following SARS-CoV-2 Infection | USA | Case reports | 2 | Age = P1 27, P2 21  Gender = P1 male and P2 female  Severity = mild | - ‘P1 presented with sudden onset confusion and aggressive behaviour. - The patient was tremulous, febrile (temperature: 102.9⁰ F) and tachycardic (heart rate: 172 beats/min) and had a small goitre on examination. - Laboratory findings were consistent with hyperthyroidism with thyroid stimulating hormone (TSH) of < 0.01 mIU/L (normal: 0.45-4.5 mIU/L), free thyroxine (T4) of > 7.8 ng/dL (normal: 0.8-1.8 ng/dL), and free triiodothyronine (T3) of 21.9 pg/mL. - Thyroid stimulating immunoglobulin (Vassiliadi et al.) and thyrotropin receptor antibody (TRAb) were both positive at 8.43 IU/L (reference range ≤0.54 IU/L) and 9.10 IU/L (reference range ≤1.75 IU/L), respectively. - Thyroid ultrasound showed a diffusely enlarged, heterogenous, hypervascular gland, consistent with Graves’ disease. - His Burch-Wartofsky score was 55 (>45 is highly suggestive of thyroid storm) prompting admission to the intensive care unit (ICU). - P2 presented with a severe headache, shortness of breath, nausea, vomiting, diarrhoea, and palpitations for 1 day. She was tachycardic (heart rate: 136 beats/min), tachypnoeic (respiratory rate: 18 breaths/min), and hypotensive (blood pressure: 84/53 mmHg). - Laboratory results revealed hyperthyroidism with TSH of <0.01 mIU/L, free T4 of >7.8 ng/dL and free T3 of 15.4 pg/mL. - Her Burch-Wartofsky score was 40 (24-44: suggestive of impending storm).’ |
| Sunetra Mondal  (Mondal et al., 2023) | Subacute thyroiditis following recovery from COVID-19 infection: novel clinical findings from an Eastern Indian cohort | India | Combined retrospective-perspective study | 160 | Age = -44  Gender = -63% women  Severity = -moderate/severe (45%) | - ‘11 patients (6.8%) were diagnosed with COVID-19-associated thyroiditis, within 3 months of infection. - Two distinct clinical subsets of post COVID-19 19 SAT—a painless variety with significant thyrotoxic symptoms, occurring more commonly in disease presenting soon after recovery from COVID-19, and a painful, less thyrotoxic variety, which predominantly occurs long after recovery from COVID-19. - On USS of the thyroid gland, findings included diffuse enlargement of the thyroid gland (n=7, 63.6%), patchy hypoechoic areas (n=11, 100%) and/or reduced vascularity (n=4, 36.4%). - All patients with painless SAT (PLSAT) and one patient with painful SAT (PFSAT) had 99mTc scan of the thyroid gland which revealed overall reduced uptake in the thyroid gland with patchy areas of uptake’ |
| Clarissa V. de Souza (de Souza et al., 2022) | Subacute thyroiditis and thyroid inflammatory nodule secondary to COVID-19: a primary care case report | Brazil | Case report | 1 | Age = 51  Gender = female  Severity = mild | - ‘Persistent odynophagia and new pain located on the left side of the lower neck. - Physical examination showed a painful goitre mostly in the left lobe, and there were no palpable lymph nodes on examin- ation. - T3 1.9 ng/mL (normal range [NR] from 0.8 to 2.0 ng/mL), T4 17.9 μg/dL (NR from 4.6 to 12.0 μg/dL), free T4 3.32 ng/dL (NR from 0.93 to 1.70 ng/dL), TSH 0.03 μmUI/L (NR from 0.27 to 4.20 μmUI/L), and CRP 93.63 mg/L (NR <5.0 mg/L). - Anti-thyroglobulin and anti-TPO antibodies were 16.8 UI/mL (NR from <115 UI/mL) and <9 UI/mL (NR <34 Ul/mL), respectively. - Thyroid USS revealed a solid nodule in the left lobe occupying the entire left lobe, measuring 4.3 × 1.6 cm, larger in width than in height, with well-defined margins and without calcification, with discrete central and peripheral vascularization. - Lead to the diagnosis of inflammatory nodule secondary to SAT.’ |
| David Tak Wai Lui (Lui, 2021) | Thyroid Dysfunction in Relation to Immune Profile, Disease Status and Outcome in 191 Patients with COVID-19 | Hong Kong | Prospective study | 191 | Age = mean 53.5 ± 17.2 years  Gender = 99 males and 92 females  Severity = 161 mild, 24 moderate and 6 severe | - ‘The most common symptoms were fever (n=96, 66.7%), cough (n=84, 58.3%), sore throat (n=59, 41.0%), diarrhea (n=37, 25.7%), and anosmia and/or ageusia (n=30, 20.8%). None of the patients presented with overt symptoms of thyroiditis such as neck pain or signs and symptoms of thyrotoxicosis. 21 patients (11.0%) presented with tachycardia. - The median TSH of the cohort was 1.20 mIU/L (IQR: 0.78–1.70) - Abnormal TFTs (defined by TSH, fT4 or fT3 out of reference ranges) were observed in 25 patients (13.1%). 14 patients (7.3%) had features of thyrotoxicosis, defined as low TSH and/or raised fT4. - 10 patients had isolated low TSH levels and another 10 patients with isolated low fT3 levels.’ |
| Runmei Zou (Zou et al., 2020) | Euthyroid Sick Syndrome in Patients With COVID-19 | USA | Retrospective cohort study | 149 (41 with ESS) | Age = median – 58  Gender = 14 male and 27 female  Severity = mild-severe | - ‘The most common symptoms in the ESS group were fever [39 (95.12%)], fatigue [18 (43.90%)], cough [36 (87.80%)], shortness of breath [25 (60.98%)], expectoration [20 (48.78%)], and anorexia [21 (51.22%)]. - Patients with ESS were older and more female dominated. - Furthermore, patients with ESS tended to have a higher prevalence of fever and shortness of breath compared to non-ESS patients. - COVID-19 patients with ESS had a significantly higher prevalence of severe events.’ |
| Weibin Wang (Wang et al., 2020) | Thyroid function abnormalities in COVID-19 patients | China | Retrospective study | 84 | Age = mean 57.3 ± 14.5 years old  Gender = 53 male, 31 female  Severity = 21 mild/moderate and 63 severe | - ‘TT3 in COVID-19 patients (1.02 ± 0.32 nmol/L) was lower than that in healthy subjects. - TSH level decreased by a lower extent (0.62 ± 0.62 mIU/L) as compared to non- COVID-19 patients (1.10 ± 0.84 mIU/L) and healthy subjects (1.56 ± 1.02 mIU/L) (p < 0.001) - TT3 and TSH were positively correlated, instead of negatively related, in COVID-19 patients. - Abnormal thyroid dysfunction was more common in severe cases (47/52, 90.4%) than mild/moderate cases (16/32, 50%).’ |
| Georgios Tsivgoulis (Georgios Tsivgoulis, 2021) | Hypothyroidism is associated with prolonged COVID-19-induced anosmia: a case–control study | Greece | Prospective case control study | 12 | Age = 26-53  Gender = 3 males, 9 females  Severity = mild disease | - ‘significant correlation between hypothyroidism and prolongation of smell dysfunction in COVID-19 patients. - this continuation of anosmia, is due to direct virus injury to the thyroid and olfactory nerve. - Therefore, SARS-CoV-2 would affect the development of these neurons ultimately leading to COVID-19 induced anosmia’ |
| Vesselina Yanachkova (Vesselina Yanachkova, 2023) | Thyroid dysfunction as a long-term post-COVID-19 complication in mild-to-moderate COVID-19 | Bulgaria | Observational prospective study | 113 | Age = median age 43 years  Gender = 78 female, 35 male  Severity = mild and moderate | - ‘61.1% had abnormal thyroid function tests after 2 months after SARS-CoV-2 - the most common thyroid dysfunction was subclinical hypothyroidism, followed by subclinical hyperthyroidism and overt hypothyroidism accounting for 78.3%, 13% and 8.7%, respectively. - Univariate logistic regression analysis demonstrated that moderate severity of COVID-19 was significantly associated with a higher risk of thyroid dysfunction. - Moderate severity of COVID-19 was also significantly associated with a higher need for levothyroxine treatment.’ |
